# Evidence on the effectiveness of community-based primary health care in improving HIV/AIDS outcomes for mothers and children in low- and middle-income countries: Findings from a systematic review

**DOI:** 10.7189/jogh.11.11001

**Published:** 2021-07-10

**Authors:** Ivy Mushamiri, Wintana Belai, Emma Sacks, Becky Genberg, Sundeep Gupta, Henry B Perry

**Affiliations:** 1 Department of Epidemiology, Mailman School of Public Health, Columbia University, New York, New York, USA; 2 Department of International Health, Division of Health Systems, Bloomberg School of Public Health, Johns Hopkins University, Baltimore, Maryland, USA; 3 Department of Epidemiology, Bloomberg School of Public Health, Johns Hopkins University, Baltimore, Maryland, USA; 4 University of California at Los Angeles, Los Angeles, California, USA

## Abstract

**Background:**

The effectiveness of community-based primary health care (CBPHC) interventions in low- and middle-income countries (LMICs), especially for maternal, neonatal and child health, is well established. However, there has not been a systematic review of the literature on the effectiveness of CBPHC on HIV outcomes derived from rigorous assessments of primary studies. Using peer-reviewed studies of randomized interventions or those containing a specified control group and directly measuring clinical HIV outcomes, we provide evidence for the effectiveness of CBPHC on HIV outcomes for mothers and children in low- and middle-income countries (LMICs).

**Methods:**

Eligibility criteria included studies assessing the effectiveness of community-based HIV interventions with or without a facility-based component, or multiple integrated projects, with outcome measures defining an aspect of HIV health status such as the utilization of prevention or health care services, nutritional status, serious morbidity (including clinical measures of HIV progression) or mortality of children aged five or younger and pregnant women. Articles published through June 3, 2020 were identified by searching four databases. The type of community-based projects implemented, the implementors, and the implementation strategies of each program were identified and the impact on HIV-related outcomes assessed.

**Results:**

The search yielded 10 537 articles; 4881 underwent title and abstract screening after removing duplicates. Of these, 117 studies qualified for full-text screening; only 22 were included in the final analysis. Most studies showed that community-based interventions improved HIV prevention and treatment outcomes compared to facility-based approaches alone. Each study had at least one statistically significant HIV-related outcome; the non-significant outcomes found in six of the 22 studies were mostly not related to HIV programming. Most interventions were implemented by community health workers; other implementers were government workers, community members, or research staff. Strategies used included peer-to-peer education, psychosocial support, training of community champions, community-based follow-up care, home-based care, and integrated care.

**Conclusions:**

CBPHC strategies are effective in improving population-based, HIV-related health outcomes for mothers and children, especially in combination with facility-based approaches. However, there is a need to assess the scalability of such interventions and integrate them into existing health systems to assess their impact on the HIV pandemic in more routine settings.

There are currently 38 million people living with HIV (PLHIV) according to recent estimates [1]. Low- and middle-income countries (LMICs) bear much of the burden of the epidemic, with sub-Saharan Africa accounting for about two-thirds of all PLHIV worldwide [1]. In 2019, only 67% of PLHIV had access to antiretroviral therapy (ART), which translates to 68% of adults with HIV aged 15 years or older, 53% of children with HIV aged 14 or younger and 85% of HIV-positive pregnant women [1,2]. Sub-Saharan Africa is disproportionally represented in vertical HIV transmissions globally; 90% of the 1.4 million pregnant women living with HIV resided in sub-Saharan Africa in 2018 and accounted for 85% of all vertical HIV transmissions [3]. Even though there has been a steady increase in the number of people accessing ART [2], treatment for HIV-positive women and children still falls short of the UNAIDS 90-90-90 goals for 2020 (upgraded to 95-95-95 goals for 2030), which aim to end the AIDS epidemic by 2030 by having 95% of all HIV positive people know their status, 95% of those knowing their status be on sustained ART and 95% of those on ART be virally suppressed [4-6]. Prevention of mother to child transmission (PMTCT) of HIV coverage in sub-Saharan Africa still stood at 79% in 2018 [3]. There is an urgent need to address the gap in HIV prevention and treatment in the context of maternal, neonatal, and child health (MNCH).

The large gap in the number of people with and without access to treatment shows that new approaches are needed to scale up existing evidence-based interventions to reach all PLHIV with life-saving treatment. As ART coverage increased over the years, many health systems in LMICs became overwhelmed by the volume of people requiring care, resulting in overcrowding of health facilities and insufficient staff to meet the rising demand [7,8]. Decentralized and “task-shifting” approaches were necessary to meet the needs of patients who did not have easy access to health facilities [7]. We can reasonably assume that those with the least access have not been reached by facility-based services and that new strategies are needed for them specifically. Additionally, as HIV becomes more of a chronic condition, long-term management becomes more necessary and feasible at the community level.

The Declaration of Alma-Ata of 1978 highlighted primary health care as the most effective way for governments to provide universal health care to their populations in an equitable way [9]. This declaration envisioned primary health care going beyond the provision of health services at the health facility level to the community level in order to make care accessible to all. Primary health care at the community level is administered mainly through the utilization of community health workers (CHWs), and also includes outreach, community organizations, health posts and other activities outside of health facilities [10]. Evidence-based interventions for HIV were originally developed and piloted in tertiary care centers and later in community hospitals and health centers, but with increasing experience are now, in many cases, deliverable at the community level. Community-based primary health care (CBPHC), which is an approach to bring preventive and curative health services beyond the health facility level to the community level [11,12], can thus be effective in meeting the gap in access to HIV services.

WHO provided task-shifting guidelines in 2007 and identified hundreds of tasks that could be “shifted” from health facility staff to CHWs [13]. These tasks focus on HIV prevention, care and treatment efforts, including health education, community mobilization, health administration, HIV testing and counseling, ART dispensing, adherence counseling, clinical management, psychosocial support, and tracing of patients lost to follow-up (LTFU) [13]. WHO recommends that these tasks be integrated with MNCH interventions and tied to existing public health infrastructures to provide comprehensive care to women and children in low-income settings.

There are a few systematic assessments of the effect of community-based approaches on HIV outcomes in the literature [14-17]. However, none of the systematic reviews or meta-analyses conducted on this subject have focused on MNCH interventions in LMICs [14-17]. There remains a gap in the assessment of how CBPHC approaches specific to MNCH have affected HIV outcomes in resource-limited settings.

We conducted a systematic review to assess the evidence of the effectiveness of CBPHC on HIV outcomes at the population level in the context of MNCH in LMICs. This review complements a previously published review of the effectiveness of community-based approaches to improving MNCH [10-12,18-23], but the interventions included in that review did not include studies related to HIV prevention and treatment.

## METHODS

The methodology for this review used the same processes as those in a previously published series of reviews assessing evidence of the effectiveness of CBPHC to improve MNCH in LMICs [11]. Briefly, we conducted a search on Pubmed, Embase, Scopus and Ovid Global Health databases for assessments of CBPHC on HIV/AIDS outcomes in the context of MNCH in LMICs. Key terms for “HIV,” “AIDS,” “maternal health,” “child health,” “community health,” “developing countries,” and related terms were identified to create a search query (see Appendix S1 in the **Online Supplementary Document**). We searched the above-mentioned databases for any articles published in peer-reviewed journals any time through June 3, 2020. Articles were uploaded into Covidence software (Veritas Health Innovation, Melbourne, Australia) and screened separately by two members of the study team, and a third member acted as a tiebreaker. Covidence software blindly assessed inter-rater agreement between reviewers at each stage of the review process. The inter-rater reliability/Cohen’s Kappa statistic for title and abstract screening and full-text screening was 0.97/0.50 and 0.96/0.87, respectively, which showed substantial agreement [24]. All procedures were conducted according to Preferred Reporting Items for Systematic reviews and Meta-Analyses (PRISMA) guidelines [25-28].

Articles were eligible for inclusion in the assessment if they met the following criteria:

1. Provided primary data for assessment of the effectiveness of CBPHC on HIV outcomes;
2. Were published in a peer-reviewed journal;
3. Included a comparison group in the design or compared outcomes before and after an intervention;
4. Involved an intervention intended to improve MNCH (i.e., conducted in a geographically defined population of mothers or children of all age-groups up to 17 years);
5. Included interventions that took place outside of a health facility;
6. Measured one of the following changes in an HIV outcome:
  - Changes in the population coverage of one or more evidence-based interventions including utilization of prevention or health care services (such as HIV testing, linkage, retention in care, or adherence to medication)
  - Stage of pregnancy at enrollment in care or age at enrollment of children
  - Changes in HIV incidence
  - Changes in mortality (HIV-related or all-cause mortality)
  - Changes in serious morbidity, including clinical measures of HIV progression (such as CD4 count, viral load, or WHO HIV staging).

Articles retrieved from the search were excluded if they were: review articles; program evaluations that were not peer-reviewed; not an experimental design, lacked a comparison group, or lacked baseline data; conducted exclusively on men or sub-populations that did not meet the criteria of MNCH; conducted in a high-income country as defined by the World Bank; exclusively implemented at a health facility; did not measure any of the HIV outcomes defined above, or only measured HIV-related risk behavior or knowledge.

We defined CBPHC as a health intervention with a community component aimed to improve the health of a geographically defined population, with services taking place partially or entirely in the community, including through outreach services based outside a health facility. CBPHC interventions included: health communication with communities; social mobilization and community involvement; and health care provision in communities, including preventive services [11].

Data extraction forms prepared for the previous comprehensive review on MNCH [11] were used for HIV outcome assessments, as the form already included a section on HIV (copies of the data extraction forms are available from the corresponding author upon request). Two independent reviewers completed a data extraction form for each article that qualified for the final assessment, and a third reviewer resolved any conflicts and summarized the data in a final data extraction form. The extraction form also included questions on the quality of the article. The final summative data containing specified variables were transferred into Microsoft Excel software. The evidence from the literature was then synthesized and summarized. The review assessed the kind of projects implemented, the outcomes of the projects, implementation strategies, and implications of the findings.

## RESULTS

### Study selection

**Figure 1:**
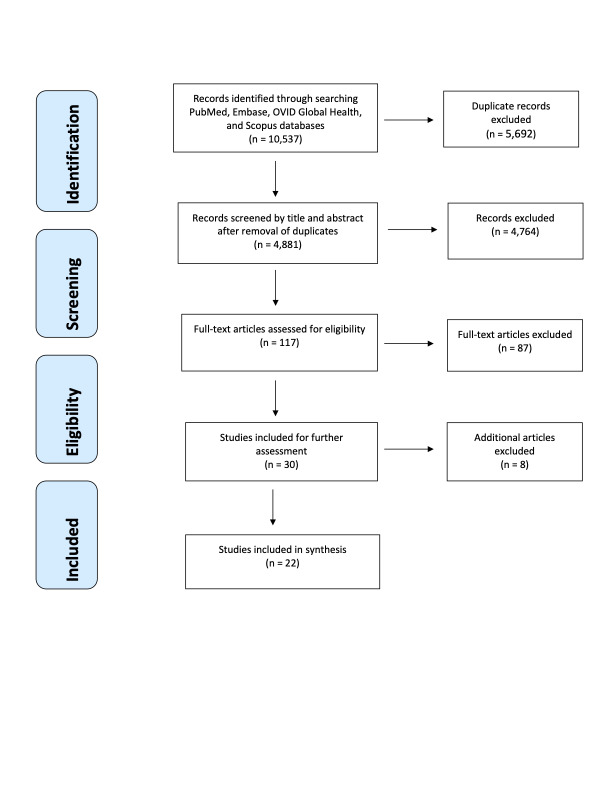
PRISMA flow diagram for systematic review

The search yielded 10 537 articles published through June 3, 2020. Initial screening identified 5692 duplicates, which were removed; 4881 studies were screened by title and abstract. Of these, 117 studies were assessed for eligibility in the full-text screening phase, of which 87 studies were excluded because they did not meet one or more of the eligibility criteria, leaving 30 articles for the data extraction phase. An additional 8 articles were removed after data extraction following a stricter assessment of the eligibility criteria, leaving 22 studies that were included for final assessment. **Figure 1** shows the PRISMA flow diagram, indicating the sequence of inclusion and exclusion of articles.

### Strength of study designs and study quality

The study designs utilized in the included studies are shown in **Table 1****.** Most of the studies had a strong design. Nine of the studies were randomized control trials (RCTs) (with randomization occurring at the cluster level for seven of them) and five were cohort studies with a control or comparison area. Seven of the studies had a pre-test/post-test design with no comparison area and one was cross-sectional assessment which compared MNCH HIV outcomes from the survey to a previous nationally representative survey conducted a few years before widespread implementation of the community-based intervention (Ethiopian Demographic Health Survey) [29]. Most of the studies were conducted with small samples (fewer than 5000 participants). They were also conducted for short periods of time (mostly 1-2 years) and involved programs with a small number of interventions. When assessed for quality, seven were rated high or exceptional quality, 11 were rated good quality and four rated poor quality.

**Table 1:** Strength of study designs

Study design	Number of studies using this design
Randomized controlled trial	9
Pre-test/post-test with no control/comparison area	7
Cohort study with control/comparison area	5
Cross-sectional with control/comparison area	1
**Total**	**22**

### Summary of studies included

**Table 2****,** **Table 3** and **Table 4** summarize the characteristics of the 22 studies included in the review [29-50]. All but two of the studies were from sub-Saharan Africa, with one study from Guatemala and another from India. The study period ranged from 2004 up to 2016. No studies were published before 2012.

**Table 2:** Summary of studies included assessing maternal health outcomes

Author and year	Country	Study period	Description of community-based intervention			Study methods			Statistically significant outcome(s)*
			**Type of persons used to implement intervention(s)**	**Intervention implementation strategy**	**Intervention**	**Outcome(s) measured**	**Measurement of effect (study design)**	**Was there a comparison with only clinic-based care?**
Audet et al, 2016 [30]	Mozambique	2012-2014	TBAs; male champions (trained counselors)	TBAs trained and supported to identify pregnant women and their partners, promote ANC, facility delivery, and PNC uptake; male champions conducted peer-to-peer education, promoted male partner engagement and accompanied couples to ANC clinic	Integration of TBAs into health system; recruitment of male champions to facilitate partner engagement in ANC services; provision of “male-friendly” clinical environments; provision of couples’ joint HIV testing and counseling	% of male accompaniment at to ANC visits	Pre-post assessment without comparison group	No	More men accompanied partners to 1st ANC
						% male partners tested for HIV during ANC			More male partners tested for HIV during ANC
						% women testing for HIV during ANC			More women tested for HIV during ANC
						% women attending 3 ANC appointments			More women attended 3 ANC appointments
						% facility deliveries			More women delivered at a health facility
Ezeanolue et al, 2015 [31]	Nigeria	2013-2014	Priests, trained, church-based volunteer health advisers, study staff, laboratory technicians	Health promotion to women attending church services and baby showers; male partner engagement; follow-up care; integrated care	Promotion of HIV testing, facility-based care for pregnant women and male partner engagement during church services; on-site integrated laboratory testing and counseling for HIV and other illnesses at church-organized baby showers	% of women tested for HIV	Cluster-randomized trial comparing intervention and standard of care (control) group	Yes	More women tested for HIV
						% of HIV-positive women linked to care			More women linked to care
						% of HIV-positive women accessing care and receiving ART			More women accessed care and were on ART during pregnancy
Fatti et al, 2016 [32]	South Africa	2009-2012	Community-based support workers (provide ART initiation and adherence counseling)	Psychosocial support to aid ART initiation and adherence; health promotion; home-based care; integrated care; follow-up care	Community-based ART initiation and adherent support for HIV-positive mothers and infants during ANC and PNC	Risk of not initiating ART antenatally	Cohort study comparing intervention group with non-intervention (control) group	Yes	Women had reduced risk of not initiating ART antenatally
						Risk of not initiating zidovudine (ZVD) for PMTCT			Women had reduced risk of not initiating ZVD for PMTCT
						% women with a stillbirth			Women had fewer stillbirths
						Time to initiate ART after 1st ANC visit			Women initiated ART with shorter delay after 1st ANC visit
						ART coverage at delivery			Women had greater ART coverage at delivery
Medhanyie et al, 2012 [29]	Ethiopia	2009	Health Extension Workers (HEWs)	Integrated care; comprehensive care; home-based care	Use of government-paid front-line health workers (HEWs) to provide comprehensive primary health and maternal health care	% of women testing for HIV	Cross-sectional study comparing intervention to a previous national survey (before deployment of HEWs)	No	More women tested for HIV
						% women utilizing ANC services			More women utilized ANC services
						% women utilizing family planning services			More women utilized family planning services
Nance et al, 2017 [33]	Tanzania	2014-2016	CHWs	Psychosocial support to aid adherence, engagement and retention in care; health promotion; home-based care; integrated care; follow-up care	Community-based ART initiation and adherence, retention in care, birth planning and integrated care support for HIV-positive mothers for PMTCT	% women adhering to ART post-partum	Cluster-randomized trial comparing intervention and standard of care (control) group	Yes	More women adhered to ART among sites with greatest fidelity to intervention only
Nyamathi et al, 2019 [34]	India	2014	CHWs	Psychosocial support to aid adherence; life-skills training; health promotion; nutrition education; integrated care	Community-based enhanced nutrition education and enhanced nutrition supplements for women living with HIV currently on ART	Increase in CD4+ T cell counts	Cluster-randomized trial comparing interventions (3 arms) and control group	No	More women had improved CD4+ T cell counts
						Increase in BMI			More women had improved BMI
						Likelihood of anemia recovery			More women recovered from anemia
Rossouw et al, 2019 [35]	South Africa	2015	CHWs	Psychosocial support; material support; health promotion; health education; home-based care; integrated care; follow-up care	Incentive package and home-visiting program by CHWs for additional psychosocial support and health education	Likelihood of making >4 ANC visits	Randomized controlled trial	Yes	More women made >4 ANC visits
						Likelihood of making 1st ANC visit before 5^th^ months of gestation			More women made 1st ANC visit before 5th months of gestation
Smith et al, 2015 [36]	Guatemala	2012-2013	Health promoters and traditional midwives	Integrated care; outreach services	Outreach teams provided integrated point-of-care (POC) antenatal screening for syphilis, hepatitis B and HIV	ANC coverage	Pre-post assessment without comparison group	No	ANC coverage increased
						% women screened for HIV			More women were screened for HIV
Vogt et al, 2015 [37]	Zimbabwe	2010-2013	CHWs trained to do defaulter tracing	Home-based care; follow-up care	Defaulter tracing of pregnant women and their newborns for PMTCT	% women retained in HIV care (did not drop out of care) at infant nevirapine (NVP) initiation date	Pre-post assessment without comparison group	No	More women were retained in care at infant NVP initiation
						% women retained in HIV care at infant cotrimoxazole (CTX) initiation date			More women were retained in care at infant CTX initiation
						% women retained in HIV care at infant HIV testing date			More women were retained in care at the time of infant HIV testing
Wangalwa et al, 2012 [38]	Kenya	2008-2010	CHWs, community health extension workers (CHEWs) and community health committees (CHCs)	Home-based care; integrated care; comprehensive care; health promotion	Community-based health system made up of CHWs, CHEWs and CHCs to promote maternal and child health	% women attending 4 or more ANC appointments	Pre-post assessment without comparison group	No	More women made 4 or more ANC visits
						% deliveries by skilled birth attendants			More deliveries by skilled birth attendants were made
						% women receiving malaria prevention treatment during pregnancy			More women received malaria prevention treatment during pregnancy
						% women tested for HIV during pregnancy			More women tested for HIV during pregnancy
						% mothers exclusively breastfeeding			More mothers breastfed exclusively

ANC – antenatal care, ART – antiretroviral therapy, PMTCT – prevention of mother-to-child transmission (of HIV), PNC – post-natal care, BMI – body mass index, IYCF – infant and young child feeding, WASH – water, sanitation, and hygiene, POC – point-of-care, CHW – community health worker, CHEW – community health extension worker, HEW – health extension worker, CHC – community health committee, TBA – traditional birth attendant, ZDV – zidovudine, NVP – nevirapine, CTX – cotrimoxazole

*5% significance level.

**Table 3:** Summary of studies included assessing child health outcomes

Author and year	Country	Study period	Description of community-based intervention			Study methods			Statistically significant outcome(s)*
			**Type of persons used to implement intervention(s)**	**Intervention implementation strategy**	**Intervention**	**Outcome(s) measured**	**Measurement of effect (study design)**	**Was there a comparison with only clinic-based care?**
Ahmed et al, 2015 [39]	Malawi	2007-2011	CHWs	Case finding (HIV testing and counseling); community engagement; health promotion; follow-up care; integrated care; home-based care	Identification and enrollment of HIV-exposed and HIV-infected infants and children	Age at enrollment in HIV programs (of HIV+ infants and children)	Pre-post assessment without comparison group	No	Age at enrollment in care decreased
Dahinten et al, 2016 [40]	Zambia	2013-2014	CHWs	Health promotion	Use of special method for dosing HIV exposed infants with ART (Pratt Pouch)	% of HIV-exposed infants receiving medication within 3 d of birth	Pre-post assessment without comparison group	No	More HIV-exposed infants were medicated within 3 d of birth
Fatti et al, 2014 [41]	South Africa	2004-2010	Patient advocates (community-based adherence supporters) and social workers	Psychosocial support to aid adherence; health promotion; home-based care; integrated care; follow-up care	Community-based adherent support for children on ART	% of children receiving ART with adequate viral suppression	Cohort study comparing intervention with non-intervention (control) group	Yes	More children who were receiving ART were virally suppressed at any time point during treatment
Ferrand et al, 2017 [42]	Zimbabwe	2013-2015	CHWs	Psychosocial support to aid adherence, engagement and retention in care; health promotion; home-based care; integrated care; follow-up care	Support to caregivers of children and adolescents newly diagnosed HIV to aid adherence, engagement and retention in care	Mortality of children in the program	Randomized trial comparing intervention and standard of care (control) group	Yes	Fewer children died
						% of children with adequate viral suppression			More children were virally suppressed
Grimwood et al, 2012 [43]	South Africa	2004-2009	Patient advocates (community-based support workers); treatment buddies (adherence supporters); social workers	Psychosocial support to aid adherence, engagement and retention in care; health promotion; home-based care; integrated care; follow-up care	Support to caregivers of children with HIV to aid adherence, engagement and retention in care	Mortality of children in the program	Cohort study comparing intervention with non-intervention (control) group	Yes	Fewer children died
						% children retained in HIV care after 3 y of ART			More children were retained HIV in care
Gupta et al, 2013 [44]	Rwanda	2007-2010	CHWs and social workers	Psychosocial support to aid adherence; health promotion; home-based care; integrated care; follow-up care	Community-based ART adherence, nutritional and sanitation support and integrated care for HIV-positive mothers and their infants (including PMTCT)	Mortality of children in the program	Pre-post assessment without comparison group	No	Fewer children died
						% of infants retained on ART at 18 mo			More children were retained in HIV care
Prendergast et al, 2019 [45]	Zimbabwe	2012-2015	CHWs	Psychosocial support to aid adherence, engagement and retention in care; health promotion; home-based care; integrated care; follow-up care	Community-based enhanced infant and young child feeding (IYCF) and improved water, sanitation, and hygiene (WASH) education and support for HIV-positive pregnant women	Increase in mean length for age	Cluster-randomized trial comparing interventions (3 arms) and control group	No	More children had improved mean length for age
						Increase in hemoglobin levels			More children had improved hemoglobin levels
						% children stunted			Fewer children were stunted
						% children with anemia			Fewer children had anemia
Thurman et al, 2016 [46]	South Africa	2014	Community-based care workers and social workers	Psychosocial support; socioeconomic support (material support and social services); home-based care; integrated care; health promotion	Home-visitation programs to meet the unique needs of each family with orphaned or vulnerable children and promote HIV testing	% children tested for HIV	Retrospective cohort study comparing intervention with non-intervention (control) group	Yes	More children were tested for HIV

ANC – antenatal care, ART – antiretroviral therapy, PMTCT - prevention of mother-to-child transmission (of HIV), PNC - post-natal care, BMI – body mass index, IYCF – infant and young child feeding, WASH – water, sanitation, and hygiene, POC – point-of-care, CHW – community health worker, CHEW – community health extension worker, HEW – health extension worker, CHC – community health committee, TBA – traditional birth attendant, ZDV – zidovudine, NVP – nevirapine, CTX – cotrimoxazole

*5% significance level.

**Table 4:** Summary of studies included assessing maternal and child health outcomes

Author and year	Country	Study period	Description of community-based intervention			Study methods			Statistically significant outcome(s)*
			**Type of persons used to implement intervention(s)**	**Intervention implementation strategy**	**Intervention**	**Outcome(s) measured**	**Measurement of effect (study design)**	**Was there a comparison with only clinic-based care?**
Aliyu et al, 2016 [47]	Nigeria	2013-2014	Peer male champions; trained midwives	Peer-to-peer education; community mobilization; male partner involvement in PMTCT care	Task-shifting to trained midwives, point-of-care CD4 count testing, integrated care (at facility level); male partner and community engagement (at community level)	% of HIV-infected women initiating ART	Cluster-randomized trial comparing intervention and standard of care (control) group	Yes	Mothers more likely to initiate ART while pregnant
						% of women and infants retained on ART at 6 weeks post-partum			Mothers and their infants more likely to remain on treatment
						Incidence of HIV in HIV-exposed infants			Infants had lower Incidence of HIV infection
Mushamiri et al, 2015[48]	Kenya	2010-2013	CHWs	mHealth; follow-up care; home-based care	Use of a CHW-centered mHealth tool to track women for ANC and PNC appointments for PMTCT	% women attending 4 or more ANC appointments	Retrospective cohort study comparing intervention with non-intervention (control) group	Yes	More women attended 4 or more ANC visits
						% women attending 6 or more post-partum baby follow-up visits			More women made 6 or more post-partum baby follow-up visits
						% vertical HIV transmission rate			Fewer HIV+ women vertically transmitted HIV to their babies
Rotheram-Borus et al, 2014 [49]	South Africa	2009-2010	CHWs	Integrated care; comprehensive care; home-based care	Home-visiting program by CHWs trained as generalists to provide maternal and child health and PMTCT support	% mother-infant pairs scoring high on overall health on 32 measures pertaining to HIV-related prevention, child health, health care, depressive symptoms and social networks	Cluster-randomized trial comparing intervention and standard of care (control) group	Yes	Mother-infant pairs had better overall health
						% mothers using condoms during sexual encounters			More mothers used condoms during sexual encounters
						% infants not undernourished according to weight-for-age measures			More infants were not undernourished according to weight-for-age measures
						% mothers exclusively breastfeeding for 6 mo			More mothers breastfed exclusively
						% mothers breastfeeding for longer			Mothers breastfed for longer
						% infants who did not have low birth weight			More infants did not have low birth weight
						% infants with normal growth according to measurers of head-circumference-for-age at 6 mo			More infants had normal growth according to measurers of head-circumference-for-age
						% infants with improved cognitive development at 18 mo			More infants had improved cognitive development
Tomlinson et al, 2014 [50]	South Africa	2008-2010	CHWs	Psychosocial support; home-based care; integrated care; health promotion	Integrated home-based package for maternal and child health and PMTCT	% mothers exclusively breastfeeding	Cluster-randomized trial comparing intervention and control group	Yes	More mothers breastfed exclusively
						% infants with increased weight-for-age			More infants had increased weight-for-age
						% infants with increased length-for-age			More infants had increased length-for-age
						% women taking infants to clinic during 1st week of life			More women took infants to clinic during 1st week
						% women making preparations for birth			More women made preparations for birth
						% women with knowledge of newborn danger signs			More women knew newborn danger signs

ANC – antenatal care, ART – antiretroviral therapy, PMTCT – prevention of mother-to-child transmission (of HIV), PNC – post-natal care, BMI – body mass index, IYCF – infant and young child feeding, WASH – water, sanitation, and hygiene, POC – point-of-care, CHW – community health worker, CHEW – community health extension worker, HEW – health extension worker, CHC – community health committee, TBA – traditional birth attendant, ZDV – zidovudine, NVP – nevirapine, CTX – cotrimoxazole

*5% significance level.

### Evidence for effectiveness of community-based approaches

All 22 studies included in the final assessment showed statistically significant improvement in at least one HIV outcome (**Table 2****,** **Table 3****,** **Table 4** and **Table 5**). Out of the 22 studies, 12 (55%) compared HIV outcomes for community-based approaches with those from facility-based approaches only and they all showed superior results achieved through combined community- and facility-based programming.

**Table 5:** HIV program outcome indicators measured

Indicator	Number of studies that assessed this indicator	Number of studies that reported a statistically significant favorable outcome on this indicator	Number of studies not showing a statistically significant favorable outcome on this indicator
% of women (or pregnant women) screened for HIV	5	5	0
% of HIV+ women (or pregnant women) initiating ART treatment	5	4	1
% of HIV+ infants who were retained in treatment	3	3	0
% of HIV+ children receiving ART with adequate viral suppression	3	2	1
Time to initiate ART after first ANC visit	2	1	1
Vertical transmission rate	2	1	1
% of HIV+ women linked to care	1	1	0
% of HIV+ women (or pregnant women) entering treatment who were retained in their treatment	1	1	0
% of HIV+ women with improved CD4+ cell count	1	1	0
% of women adhering to ART during the post-partum period	1	1	0
% of women retained in care during the post-partum period	1		1
ART coverage at delivery	1	1	
% of women retained in care at delivery	1		1
% of women retained in care at infant HIV testing	1	1	0
% of women retained in care at infant ART initiation	1	1	0
% of children tested for HIV compared to non-intervention group	1	1	0
Incidence of HIV in HIV-exposed infants	1	1	0
% of HIV-exposed infants who tested HIV+ receiving ART within 3 d of birth	1	1	0
Average age of HIV+ infant/child in enrollment for ART	1	1	0

ANC – antenatal care, ART – antiretroviral therapy

**Table 5** summarizes the discrete HIV outcomes measured, each comparing a group receiving a community-based intervention to a control group or comparing the same group/community before and after the implementation of the community-based intervention. Five of the studies assessed the proportion of women (pregnant or not) screened for HIV after a community-based intervention, and all reported statistically significant positive findings. One study assessed the proportion of HIV-positive women linked to care (engaged with the formal health system by presenting for care at a health facility) and reported statistically significant positive findings. Of the five studies assessing the proportion of HIV-positive women (pregnant or not) initiating ART treatment, four found statistically significant positive findings. Two studies assessed the time to initiation of ART after the first antenatal care (ANC) visit, and one of them found a significant impact of the interventions. Other outcomes where at least one study found statistically significant findings were: proportion of HIV-positive women (pregnant or not) entering treatment who continued their treatment at the time of the next assessment; proportion of HIV-positive women with improved CD4+ cell count; proportion of women adhering to ART during the post-partum period; ART coverage at delivery; vertical HIV transmission rates; proportion of women retained in care at infant HIV testing; proportion of women retained in care at the time of infant ART initiation; proportion of children tested for HIV compared to a non-intervention group; incidence of HIV in HIV-exposed infants; proportion of HIV-exposed infants who tested HIV positive receiving ART within three days of birth. Additionally, all three of the studies that assessed proportion of HIV-positive infants who were retained in treatment (at 6 weeks post-partum, at 18 months, or after 3 years on ART) found statistically significant positive findings. Two of the three studies assessing proportion of HIV-infected children receiving ART with adequate viral suppression (at 12 months or at any point up to four years of treatment) found statistically significant positive findings. Finally, one study found that community-based approaches significantly decreased the average age of HIV-positive infant/child enrollment for ART.

Many (10/22) of the studies also reported statistically significant positive effects on other aspects of MNCH services. The following are the outcomes and in parenthesis are the number of studies for which that outcome was positive:

- ANC or postnatal care (PNC) utilization (6)
- Nutritional status of children (4)
- Birthweight (4)
- Prevalence of exclusive breastfeeding [coverage and duration] (3)
- Proportion of mothers making preparations for birth (1)
- Cognitive development (1)
- Condom use during all sexual encounters (1)
- Proportion of deliveries attended by a skilled attendant (1)
- Proportion of births taking place in facility (1)
- Family planning (FP) utilization (1)
- Knowledge of maternal danger signs (1)
- Male partner accompaniment to first ANC (1)
- Receipt of malaria prevention treatment (1)
- Utilization of clinic for newborn care (1)

There were no studies that reported unfavorable statistically significant outcomes comparing community-based interventions to facility-based interventions alone. There were six studies that reported one or more outcomes that were not statistically significant. However, all of the six studies also reported at least one HIV-related outcome that was statistically significant and favorable. Some of the outcomes that were not statistically significant were part of a broader program rather than a program that focused only on HIV (eg, syphilis testing, facility births, PNC, or use of iodized salt).

Fifteen of the 22 studies combined an HIV intervention with an integrated (more comprehensive) program of community-based services in order to promote: ANC/PNC/facility-based delivery; male partner accompaniment to facility-based care; comprehensive, home-based integrated care; FP utilization; use of iodized salt; exclusive breastfeeding; making birth preparations; knowledge of newborn danger signs; receipt of malaria-prevention treatment during pregnancy; syphilis screening; mebendazole and vitamin A administration; monthly food packages; training of mothers on the recognition and treatment of diarrheal disease, clean water preparation, FP utilization, proper use of replacement foods, nutrition, and common childhood illnesses; general maternal and child health; socioeconomic support (material support and social services); HIV or general health counseling; and referral for different health and social services. For example, the use of government-paid, front line health extension workers (HEWs) in Ethiopia to provide comprehensive primary health and maternal health care was found to have broad health benefits such as increased utilization of ANC services and FP services in addition to increased HIV testing [29].

### Implementers

**Table 6** summarizes the implementers of the interventions at community level. CHWs working exclusively on MNCH/HIV programs were involved in 12 of the 22 programs, and 10 of these programs paid or provided financial incentives to their CHWs. Nine studies also included CHWs working on integrated programs beyond HIV, of which eight utilized paid CHWs, and in some instances the CHWs working on integrated programs were supervisors of the CHWs working exclusively on HIV/MNCH programs. Other community-level implementers included community health committees, priests or church-based volunteer health advisors, patient advocates, treatment buddies or peer-support counselors, male partners, or male champions. Additionally, three studies utilized social workers, and three studies used traditional birth attendants (TBAs) or traditional midwives to implement the community-based interventions. Because of the small number of studies available for review with heterogeneous strategies and outcomes which were not designed for determining which strategy was most effective, we cannot draw any conclusions about which arrangements were most effective.

**Table 6:** Type of implementer of the intervention at community level

Type of implementer used to deliver intervention(s)	Number of studies using this type of implementer
Paid CHW (working only on MNCH/HIV)	10
Paid CHW (working on integrated program beyond HIV)	8
Social worker	4
Community-based support worker	3
TBA/traditional midwife	3
Male partner/male champion	2
Patient advocate/treatment buddy/peer-support counselor	2
Volunteer CHW (working only on MNCH/HIV)	2
Community Health Committee	1
Church-based volunteer health advisor	1
Priest	1
Volunteer CHW (working on integrated program beyond HIV)	1

MNCH – maternal, neonatal and child health, TBA – traditional birth attendant, CHW – community health worker

### Implementation strategies

**Table 7** shows the strategies used to implement the community-based interventions to improve MNCH and HIV outcomes, and **Table 8** shows the technical interventions provided in the community. Almost all (20 out of 22) of the programs conducted home visits or provided one-on-one face-to-face counseling or support at the community level. The same number of studies also integrated their programs into existing MNCH/PHC programs. Twelve of the programs traced patients LTFU or provided or follow-up care. Five of the interventions were delivered through community meetings, and one utilized mHealth techniques to aid PMTCT efforts. The technical interventions provided in the community involved HIV testing in five studies, provision of ART to mothers in two studies, and provision of ART to children in one study.

**Table 7:** Strategies used to deliver intervention

Strategy	Number of studies using this strategy
Home visits/one-on-one face-to-face counseling/support	20
Integration into existing MNCH/PHC program	20
Tracing patients LTFU/follow-up care	12
Community meetings	5
mHealth	1

MNCH – maternal, neonatal and child health, LTFU – lost to follow-up

**Table 8:** Technical intervention provided in the community

Technical intervention	Number of studies providing this intervention
HIV testing	5
Provision of ART to mother	2
Provision of ART to child	1

ART – antiretroviral therapy

## DISCUSSION

This assessment found strong evidence for a positive impact of CBPHC interventions on HIV outcomes for mothers and children in LMICs. All 22 studies assessed showed a positive statistically significant finding for one or more HIV outcomes and more than half the studies compared a community-based intervention to a facility-based only strategy and found the one incorporating community-based components to be more effective in reducing HIV-related morbidity and mortality for women and children.

The most common HIV outcomes assessed included proportion of women screened for HIV, proportion of HIV-infected women initiating ART, proportion of HIV-infected infants retained on treatment (at 6 weeks post-partum, at 18 months, or after 3 years on ART), and proportion infants on ART that were virally suppressed (at 12 months or at any point up to four years of treatment). Most studies found statistically significant positive outcomes for the CBPHC component. Most of the interventions were implemented by paid CHWs working exclusively on MNCH or HIV, while other interventions were implemented by CHWs integrated into other programs. The community-based implementors used several strategies to deliver interventions, including home visits or one-on-one counseling, integration into existing MNCH/PHC programs, tracing of patients LTFU or follow-up care, community meetings and mHealth (such as SMS reminders for clinic appointments). The technical interventions included HIV testing and provision of ART to the mother and/or the child.

Among the studies in our review, community-based approaches out-performed facility-based ones (which did not have a community component) in HIV care, most likely because they helped counter the burden of traveling to a health facility and removed hindrances to retention in the HIV care continuum [4-6]. Data from national HIV surveys indicate that most countries with generalized HIV epidemics were not on track to meet all three of the UNAIDS 95-95-95 goals by 2020 [51-53], and in Eastern and Southern Africa, the region with the highest burden of HIV in the world, the 95-95-95 estimate was 87-72-65 in 2019 [1]. Community-based approaches such as home-based HIV testing and counselling (HBHTC) have been recommended by the WHO to remove the barrier of the initial engagement with the health system by bringing care to the doorstep of diagnosed and undiagnosed persons who are HIV-infected [54]. HIV testing and counseling at the household level is one strategy that was implemented by studies in this review and was effective for improving HIV testing rates compared to purely facility-based testing programs. Although HBHTC improves HIV testing rates, linkage rates are however still very low in resource-limited settings such as many in sub-Saharan Africa, with only about a third of people linking to care after HBHTC [55,56]. Additional strategies such as tracing of patients LTFU, follow-up care, and mHealth are needed to meet and sustain these targets at a population level.

Our results are comparable to the results found in the series of reviews assessing evidence of the effectiveness of a number of non-HIV interventions provided through CBPHC to improve MNCH in LMICs [10-12,18-23]. Community-based interventions are effective in improving multiple MNCH key indicators [10-12,18-23]. Other systematic reviews specific to HIV found mixed results. A 2014 systematic review and meta-analysis of randomized trials to assess the effect of home-based interventions on African adult populations receiving ART did not find a difference in virological outcomes between standard of care and home-based interventions arms [14]. The authors concluded that given the very few trials done on the subject, there was insufficient information to make a firm conclusion [14]. A 2013 systematic review assessed the different models of community-based ART programs in sub-Saharan Africa and found that the most successful models that made treatment more accessible and affordable involved either CHWs delivering ART at home or self-formed community ART delivery groups consisting of PLHIV [15]. The authors however acknowledged the need for several conditions to be met in order for these community-based approaches to be effective: 1) the programs need to be community-driven, 2) the environment needs to be enabling and supportive to ensure that task-shifting does not compromise the quality of services provided, and 3) there need to be long-term financial commitments from national governments and international donors [15]. These proposed conditions are supported in the current review, and our findings also show the efficiency of effective integrated programs that utilize the same CHWs to administer HIV interventions at the community level alongside existing MNCH programs or interventions in other programs or disease areas. This will require a political and social environment that fosters the harmonization of CHW programs and strong government leadership to standardize the operation of the programs, avoid redundancy, and ultimately strengthen the health system.

A 2016 systematic review of interventions to improve post-partum retention of women in PMTCT and ART care found phone-based interventions such as text messaging to be the only promising intervention of those studied to improve retention in PMTCT in the first one to three months after childbirth [16]. Phone-based interventions were often provided by CHWs (text message reminders to present to the clinic for care were either sent directly to the women by CHWs or were sent to the CHWs from health facilities to urge them to follow-up on women who have not been retained in care) [16]. Phone-based (mHealth) interventions were also found to be effective in promoting engagement in care in the current review, alongside interventions involving home-based care, integrated care, follow-up care, psychosocial support and community engagement. A 2016 systematic review and meta-analysis on the effect of community-based ART dispensing (vs facility-based dispensing) on treatment engagement in LMICs found that overall, compared to facility-based programs, community-based programs had higher rates of treatment engagement but had comparable overall outcomes for clinically stable HIV-infected patients and for all patients in terms of ART adherence, virological suppression, and all-cause mortality [17]. In the current review, community-based programs that assessed levels of engagement in HIV care, serious morbidity such as clinical indicators of HIV progression (CD4 count and viral load suppression), and infant mortality had better outcomes than programs that were only facility-based.

This review has several strengths. It appears to be the first review to systematically assess the evidence of the effectiveness of CBPHC on HIV outcomes in the context of MNCH in LMICs. Only studies utilizing a rigorous methodology were eligible for inclusion in the review, ensuring that the findings had strong grounds for internal validity. Most of the studies in this review were of high or exceptional quality. This review, however, had a few limitations. The studies assessed tended to be small and of short duration, making it difficult to determine the long-term effects of the community-based interventions at a population level. Also, because of the small number and heterogeneity of studies included, we could not assess the specific strategies that resulted in the best improvement in HIV outcomes in the context of CBPHC. Finally, this review was restricted to studies conducted in LMICs, hence the findings may not be generalizable to high-income countries. Future research should include more RCTs, which allow for stronger internal validity and the inference of causality, in order to provide stronger evidence for the effectiveness of CBPHC on HIV outcomes. Outcome measures need to be more specific and measurable and the standardization of outcome indicators would make it easier to compare across studies. Studies should also be of longer duration with bigger sample sizes in order to assess the long-term effects of the community-based interventions at a population level.

## CONCLUSION

Community-based programs are effective in improving HIV outcomes for mothers and their children and, when compared with programs providing only facility-based care, adding community-based approaches can lead to more effective outcomes than facility-based ones alone. The most successful community-based interventions for HIV care and treatment employ various strategies that foster community engagement and usually include CHWs, home visits, and home-based care to remove barriers to entry into the health care system. CBPHC programs are an important addition to facility-based care in order to achieve universal health care and for improving HIV outcomes in the context of MNCH in LMICs.

## Additional material

Online Supplementary Document
