# Supplementary material for: Evidence on the effectiveness of community-based primary health care in improving HIV/AIDS outcomes for mothers and children in low- and middle-income countries: Findings from a systematic review
Source: J Glob Health. 2021 Jul 10;11:11001. doi: 10.7189/jogh.11.11001 (PMC8284540; doi:10.7189/jogh.11.11001)
Supplement: Online Supplementary Document [file jogh-11-11001-s001.pdf]

SUPPLEMENT: FINAL SEARCH TERMS SUMMARY

PubMed

Embase

Scopus

29104904 OR 29058457 OR **29036208** OR **28859083** OR **28787328 OR 28770598** OR **28544728** OR **27930611** OR **27509237** OR **27045002** OR **26906021 OR 26518590** OR **22789644 OR 23467438** OR **26475016** OR **25886279** OR **25340337** OR **25968489** OR **23467721** OR 20714273 OR 15851922 OR **24047764** OR **23202811** OR **25436818** OR **26462714** OR **24433230** OR **25571857**

Concept 1

"maternal self-administration"[tw] OR "child welfare"[MeSH Terms] OR Community health workers[mesh] OR "Community health workers"[mesh] OR "child welfare"[MeSH Terms] OR "Community Networks"[Mesh] OR "Community Health Aides"[Mesh] OR "Community Health Nursing"[Mesh] OR "Community Medicine"[Mesh] OR community network[tw] OR community networks[tw] OR community health aide[tw] OR community health aides[tw] OR community health nursing[tw] OR community medicine[tw] OR community health nurses[tw] OR community health nurse[tw] OR community health officers[tw] OR community health volunteer[tw] OR community health volunteers[tw] OR community health worker[tw] OR community health workers[tw] OR community IMCI[tw] OR community-oriented primary care[tw] OR community oriented primary care[tw] OR community volunteer[tw] OR community volunteers[tw] OR health extension workers[tw] OR integrated child development services[tw] OR Paramedical worker[tw] OR paramedical workers[tw] OR village development committee[tw] OR village development committees[tw] OR village health worker[tw] OR village health workers[tw] OR village health volunteer[tw] OR village health volunteers[tw] OR community-based nutrition programs[tw] OR community based nutrition programs[tw] OR community-based program[tw] OR community-based programs[tw] OR community-based programme[tw] OR community-based programmes[tw] OR community based programme[tw] OR community based programmes[tw] OR community involvement[tw] OR community participation[tw] OR community program[tw] OR community programs[tw] OR community programme[tw] OR community programmes[tw]

Concept 2:

"HIV"[mesh] OR "HIV"[tw] OR "AIDS"[tw] OR "HIV-1"[mesh] OR "HIV-2"[mesh] OR "Human immunodeficiency viruses"[tw] OR "HTLV-III"[tw] OR "Human Immunodeficiency Virus"[tw] OR "Acquired Immune Deficiency"[tw] OR "Acquired Immunodeficiency Syndrome"[Mesh] OR "Acquired Immunodeficiency"[tw] OR "HIV Infections"[Mesh] OR "T Lymphotropic Virus Type III Infections"[tw] OR "T-Lymphotropic Virus Type III Infection"[tw] OR "acquired immunologic deficiency"[tw] OR "Antiretroviral Therapy, Highly Active"[Mesh] OR "Antiretroviral Therapy"[tw] OR HAART[tw]

Concept 3: LMIC

(“emerging country”[all fields] OR “emerging countries”[all fields] OR “emerging nation”[all fields] OR “emerging nations”[all fields] OR “emerging population”[all fields] OR “emerging populations”[all fields] OR "developing country"[tiab] OR "developing countries"[tiab] OR "developing nation"[tiab] OR

"developing nations"[tiab] OR "developing population"[tiab] OR "developing populations"[tiab] OR "developing world"[tiab] OR "less developed country"[tiab] OR "less developed countries"[tiab] OR "less developed nation"[tiab] OR "less developed nations"[tiab] OR "less developed population"[tiab] OR "less developed populations"[tiab] OR "less developed world"[tiab] OR "lesser developed country"[tiab] OR "lesser developed countries"[tiab] OR "lesser developed nation"[tiab] OR "lesser developed nations"[tiab] OR "lesser developed population"[tiab] OR "lesser developed populations"[tiab] OR "lesser developed world"[tiab] OR "under developed country"[tiab] OR "under developed countries"[tiab] OR "under developed nation"[tiab] OR "under developed nations"[tiab] OR "under developed population"[tiab] OR "under developed populations"[tiab] OR "under developed world"[tiab] OR "underdeveloped country"[tiab] OR "underdeveloped countries"[tiab] OR "underdeveloped nation"[tiab] OR "underdeveloped nations"[tiab] OR "underdeveloped population"[tiab] OR "underdeveloped populations"[tiab] OR "underdeveloped world"[tiab] OR "middle income country"[tiab] OR "middle income countries"[tiab] OR "middle income nation"[tiab] OR "middle income nations"[tiab] OR "middle income population"[tiab] OR "middle income populations"[tiab] OR "low income country"[tiab] OR "low income countries"[tiab] OR "low income nation"[tiab] OR "low income nations"[tiab] OR "low income population"[tiab] OR "low income populations"[tiab] OR "lower income country"[tiab] OR "lower income countries"[tiab] OR "lower income nation"[tiab] OR "lower income nations"[tiab] OR "lower income population"[tiab] OR "lower income populations"[tiab] OR "underserved country"[tiab] OR "underserved countries"[tiab] OR "underserved nation"[tiab] OR "underserved nations"[tiab] OR "underserved population"[tiab] OR "underserved populations"[tiab] OR "underserved world"[tiab] OR "under served country"[tiab] OR "under served countries"[tiab] OR "under served nation"[tiab] OR "under served nations"[tiab] OR "under served population"[tiab] OR "under served populations"[tiab] OR "under served world"[tiab] OR "deprived country"[tiab] OR "deprived countries"[tiab] OR "deprived nation"[tiab] OR "deprived nations"[tiab] OR "deprived population"[tiab] OR "deprived populations"[tiab] OR "deprived world"[tiab] OR "poor country"[tiab] OR "poor countries"[tiab] OR "poor nation"[tiab] OR "poor nations"[tiab] OR "poor population"[tiab] OR "poor populations"[tiab] OR "poor world"[tiab] OR "poorer country"[tiab] OR "poorer countries"[tiab] OR "poorer nation"[tiab] OR "poorer nations"[tiab] OR "poorer population"[tiab] OR "poorer populations"[tiab] OR "poorer world"[tiab] OR "developing economy"[tiab] OR "developing economies"[tiab] OR "less developed economy"[tiab] OR "less developed economies"[tiab] OR "lesser developed economy"[tiab] OR "lesser developed economies"[tiab] OR "under developed economy"[tiab] OR "under developed economies"[tiab] OR "underdeveloped economy"[tiab] OR "underdeveloped economies"[tiab] OR "middle income economy"[tiab] OR "middle income economies"[tiab] OR "low income economy"[tiab] OR "low income economies"[tiab] OR "lower income economy"[tiab] OR "lower income economies"[tiab] OR "low gdp"[tiab] OR "low gnp"[tiab] OR "low gross domestic"[tiab] OR "low gross national"[tiab] OR "lower gdp"[tiab] OR "lower gnp"[tiab] OR "lower gross domestic"[tiab] OR "lower gross national"[tiab] OR lmic[tiab] OR lmics[tiab] OR "third world"[tiab] OR "lami country"[tiab] OR "lami countries"[tiab] OR "transitional country"[tiab] OR "transitional countries"[tiab] OR Africa[tiab] OR Asia[tiab] OR Caribbean[tiab] OR West Indies[tiab] OR South America[tiab] OR Latin America[tiab] OR Central America[tiab] OR "Atlantic Islands"[tiab] OR "Commonwealth of Independent States"[tiab] OR "Pacific Islands"[tiab] OR "Indian Ocean Islands"[tiab] OR "Eastern Europe"[tiab] OR Afghanistan[tiab] OR Albania[tiab] OR Algeria[tiab] OR Angola[tiab] OR Armenia[tiab] OR Armenian[tiab] OR Azerbaijan[tiab] OR Bangladesh[tiab] OR Benin[tiab] OR Byelarus[tiab] OR Byelorussian[tiab] OR Belarus[tiab] OR Belorussian[tiab] OR Belorussia[tiab] OR Belize[tiab] OR Bhutan[tiab] OR Bolivia[tiab] OR Bosnia[tiab] OR Herzegovina[tiab] OR Hercegovina[tiab] OR Botswana[tiab] OR Brasil[tiab] OR Brazil[tiab] OR Bulgaria[tiab] OR Burkina Faso[tiab] OR Burkina Fasso[tiab] OR Upper Volta[tiab] OR Burundi[tiab] OR Urundi[tiab] OR Cambodia[tiab] OR Khmer Republic[tiab] OR Kampuchea[tiab] OR Cameroon[tiab] OR Cameroons[tiab] OR Cameron[tiab] OR Cape Verde[tiab] OR Central African

Republic[tiab] OR Chad[tiab] OR China[tiab] OR Taiwan[tiab] OR "Taiwan"[Mesh] OR Colombia[tiab] OR Comoros[tiab] OR Comoro Islands[tiab] OR Comores[tiab] OR Mayotte[tiab] OR Congo[tiab] OR Zaire[tiab] OR Costa Rica[tiab] OR Cote d'Ivoire[tiab] OR Ivory Coast[tiab] OR Cuba[tiab] OR Czechoslovakia[tiab] OR Slovakia[tiab] OR Djibouti[tiab] OR French Somaliland[tiab] OR Dominica[tiab] OR Dominican Republic[tiab] OR East Timor[tiab] OR East Timur[tiab] OR Timor Leste[tiab] OR Ecuador[tiab] OR Egypt[tiab] OR El Salvador[tiab] OR Eritrea[tiab] OR Ethiopia[tiab] OR Fiji[tiab] OR Gabon[tiab] OR Gabonese Republic[tiab] OR Gambia[tiab] OR Gaza[tiab] OR Georgia Republic[tiab] OR Georgian Republic[tiab] OR Ghana[tiab] OR Gold Coast[tiab] OR Grenada[tiab] OR Guatemala[tiab] OR Guinea[tiab] OR Guiana[tiab] OR Guyana[tiab] OR Haiti[tiab] OR Honduras[tiab] OR India[tiab] OR Maldives[tiab] OR Indonesia[tiab] OR Iran[tiab] OR Iraq[tiab] OR Jamaica[tiab] OR Jordan[tiab] OR Kazakhstan[tiab] OR Kazakh[tiab] OR Kenya[tiab] OR Kiribati[tiab] OR Korea[tiab] OR Kosovo[tiab] OR Kyrgyzstan[tiab] OR Kirghizia[tiab] OR Kyrgyz Republic[tiab] OR Kirghiz[tiab] OR Kirgizstan[tiab] OR "Lao PDR"[tiab] OR Laos[tiab] OR Lebanon[tiab] OR Lesotho[tiab] OR Basutoland[tiab] OR Liberia[tiab] OR Libya[tiab] OR Macedonia[tiab] OR Madagascar[tiab] OR Malagasy Republic[tiab] OR Malaysia[tiab] OR Malaya[tiab] OR Malay[tiab] OR Sabah[tiab] OR Sarawak[tiab] OR Malawi[tiab] OR Nyasaland[tiab] OR Mali[tiab] OR Marshall Islands[tiab] OR Mauritania[tiab] OR Mauritius[tiab] OR Agalega Islands[tiab] OR "Melanesia"[tiab] OR Mexico[tiab] OR Micronesia[tiab] OR Middle East[tiab] OR Moldova[tiab] OR Moldovia[tiab] OR Moldovian[tiab] OR Mongolia[tiab] OR Montenegro[tiab] OR Morocco[tiab] OR Ifni[tiab] OR Mozambique[tiab] OR Myanmar[tiab] OR Myanma[tiab] OR Burma[tiab] OR Namibia[tiab] OR Nepal[tiab] OR Nicaragua[tiab] OR Niger[tiab] OR Nigeria[tiab] OR Muscat[tiab] OR Pakistan[tiab] OR Palau[tiab] OR Palestine[tiab] OR Panama[tiab] OR Paraguay[tiab] OR Peru[tiab] OR Philippines[tiab] OR Philipines[tiab] OR Phillipines[tiab] OR Phillippines[tiab] OR Romania[tiab] OR Rumania[tiab] OR Roumania[tiab] OR Rwanda[tiab] OR Ruanda[tiab] OR Saint Kitts[tiab] OR St Kitts[tiab] OR Nevis[tiab] OR Saint Lucia[tiab] OR St Lucia[tiab] OR Saint Vincent[tiab] OR St Vincent[tiab] OR Grenadines[tiab] OR Samoa[tiab] OR Samoan Islands[tiab] OR Navigator Island[tiab] OR Navigator Islands[tiab] OR Sao Tome[tiab] OR Senegal[tiab] OR Serbia[tiab] OR Montenegro[tiab] OR Sierra Leone[tiab] OR Sri Lanka[tiab] OR Ceylon[tiab] OR Solomon Islands[tiab] OR Somalia[tiab] OR Sudan[tiab] OR Suriname[tiab] OR Surinam[tiab] OR Swaziland[tiab] OR Syria[tiab] OR Syrian[tiab] OR Tajikistan[tiab] OR Tadzhikistan[tiab] OR Tadjikistan[tiab] OR Tadzhik[tiab] OR Tanzania[tiab] OR Thailand[tiab] OR Togo[tiab] OR Togolese Republic[tiab] OR Tonga[tiab] OR Tunisia[tiab] OR Turkey[tiab] OR Turkmenistan[tiab] OR Turkmen[tiab] OR Tuvalu[tiab] OR Uganda[tiab] OR Ukraine[tiab] OR Uzbekistan[tiab] OR Uzbek OR Vanuatu[tiab] OR New Hebrides[tiab] OR Vietnam[tiab] OR Viet Nam[tiab] OR West Bank[tiab] OR Yemen[tiab] OR Yugoslavia[tiab] OR Zambia[tiab] OR Zimbabwe[tiab] OR Rhodesia[tiab] OR Developing Countries[Mesh] OR Africa[Mesh:NoExp] OR Africa, Northern[Mesh:NoExp] OR Africa South of the Sahara[Mesh:NoExp] OR Africa, Central[Mesh:NoExp] OR Africa, Eastern[Mesh:NoExp] OR Africa, Southern[Mesh:NoExp] OR Africa, Western[Mesh:NoExp] OR Asia[Mesh:NoExp] OR Asia, Central[Mesh:NoExp] OR Asia, Southeastern[Mesh:NoExp] OR Asia, Western[Mesh:NoExp] OR Caribbean Region[Mesh:NoExp] OR West Indies[Mesh:NoExp] OR South America[Mesh:NoExp] OR Latin America[Mesh:NoExp] OR Central America[Mesh:NoExp] OR "Atlantic Islands"[Mesh:NoExp] OR "Commonwealth of Independent States"[Mesh:NoExp] OR "Pacific Islands"[Mesh:NoExp] OR "Indian Ocean Islands"[Mesh:NoExp] OR "Europe, Eastern"[Mesh:NoExp] OR Afghanistan[Mesh] OR Albania[Mesh] OR Algeria[Mesh] OR American Samoa[Mesh] OR Angola[Mesh] OR Armenia[Mesh] OR Azerbaijan[Mesh] OR "Baltic States"[Mesh] OR Bangladesh[Mesh] OR Benin[Mesh] OR "Republic of Belarus"[Mesh] OR Belize[Mesh] OR Bhutan[Mesh] OR Bolivia[Mesh] OR Bosnia-Herzegovina[Mesh] OR Botswana[Mesh] OR Brazil[Mesh] OR Bulgaria[Mesh] OR Burkina Faso[Mesh] OR Burundi[Mesh] OR Cambodia[Mesh] OR Cameroon[Mesh] OR Cape Verde[Mesh] OR Central African Republic[Mesh] OR Chad[Mesh]] OR China[Mesh] OR Colombia[Mesh] OR Comoros[Mesh] OR Congo[Mesh] OR Costa Rica[Mesh] OR Cote d'Ivoire[Mesh] OR Cuba[Mesh] OR

Czechoslovakia[Mesh] OR Slovakia[Mesh] OR Djibouti[Mesh] OR "Democratic Republic of the Congo"[Mesh] OR "Democratic People's Republic of Korea"[Mesh] OR Dominica[Mesh] OR Dominican Republic[Mesh] OR East Timor[Mesh] OR Ecuador[Mesh] OR Egypt[Mesh] OR El Salvador[Mesh] OR Eritrea[Mesh] OR Ethiopia[Mesh] OR Fiji[Mesh] OR "French Guiana"[Mesh] OR Gabon[Mesh] OR Gambia[Mesh] OR "Georgia (Republic)"[Mesh] OR Ghana[Mesh] OR Grenada[Mesh] OR Guatemala[Mesh] OR Guinea[Mesh] OR Guinea-Bissau[Mesh] OR Guyana[Mesh] OR Haiti[Mesh] OR Honduras[Mesh] OR "Independent State of Samoa"[Mesh] OR India[Mesh] OR Indonesia[Mesh] OR Iran[Mesh] OR Iraq[Mesh] OR Jamaica[Mesh] OR Jordan[Mesh] OR Kazakhstan[Mesh] OR Kenya[Mesh] OR Korea[Mesh] OR Kyrgyzstan[Mesh] OR Laos[Mesh] OR Lebanon[Mesh] OR Lesotho[Mesh] OR Liberia[Mesh] OR Libya[Mesh] OR "Macedonia (Republic)"[Mesh] OR Madagascar[Mesh] OR Malawi[Mesh] OR Malaysia[Mesh] OR Mali[Mesh] OR Mauritania[Mesh] OR Mauritius[Mesh] OR "Melanesia"[Mesh] OR Mexico[Mesh] OR Micronesia[Mesh] OR Middle East[Mesh:NoExp] OR Moldova[Mesh] OR Mongolia[Mesh] OR Montenegro[Mesh] OR Morocco[Mesh] OR Mozambique[Mesh] OR Myanmar[Mesh] OR Namibia[Mesh] OR Nepal[Mesh] OR Nicaragua[Mesh] OR Niger[Mesh] OR Nigeria[Mesh] OR Pakistan[Mesh] OR Palau[Mesh] OR Panama[Mesh] OR Papua New Guinea[Mesh] OR Paraguay[Mesh] OR Peru[Mesh] OR Philippines[Mesh] OR "Republic of Korea"[Mesh] OR Romania[Mesh] OR Rwanda[Mesh] OR Saint Lucia[Mesh] OR "Saint Vincent and the Grenadines"[Mesh] OR Samoa[Mesh] OR Senegal[Mesh] OR Serbia[Mesh] OR Montenegro[Mesh] OR Sierra Leone[Mesh] OR Sri Lanka[Mesh] OR Somalia[Mesh] OR South Africa[Mesh] OR Sudan[Mesh] OR Suriname[Mesh] OR Swaziland[Mesh] OR Syria[Mesh] OR Tajikistan[Mesh] OR Tanzania[Mesh] OR Thailand[Mesh] OR Togo[Mesh] OR Tonga[Mesh] OR Tunisia[Mesh] OR Turkey[Mesh] OR Turkmenistan[Mesh] OR Uganda[Mesh] OR Ukraine[Mesh] OR Uzbekistan[Mesh] OR Vanuatu[Mesh] OR Vietnam[Mesh] OR Yemen[Mesh] OR Yugoslavia[Mesh] OR Zambia[Mesh] OR Zimbabwe[Mesh] OR “Southern African Development Community”[all fields] OR “East African Community"[all fields] OR “West African Health Organisation"[all fields] OR “Sub Saharan Africa "[all fields] OR “SubSaharan Africa "[all fields])

PubMed Search:

("HIV"[mesh] OR “HIV”[tw] OR "AIDS"[tw] OR “HIV-1”[mesh] OR “HIV-2”[mesh] OR “Human immunodeficiency viruses”[tw] OR “HTLV-III”[tw] OR "Human Immunodeficiency Virus"[tw] OR "Acquired Immune Deficiency"[tw] OR “Acquired Immunodeficiency Syndrome”[Mesh] OR “Acquired Immunodeficiency”[tw] OR "HIV Infections"[Mesh] OR “T Lymphotropic Virus Type III Infections”[tw] OR “T-Lymphotropic Virus Type III Infection”[tw] OR "acquired immunologic deficiency"[tw] OR "Antiretroviral Therapy, Highly Active"[Mesh] OR "Antiretroviral Therapy"[tw] OR HAART[tw])

AND

("maternal self-administration"[tw] OR "child welfare"[MeSH Terms] OR Community health workers[mesh] OR "Community health workers"[mesh] OR "child welfare"[MeSH Terms] OR "Community Networks"[Mesh] OR "Community Health Workers"[Mesh] OR "Community Health Nursing"[Mesh] OR "Community Medicine"[Mesh] OR community network[tw] OR community networks[tw] OR community health aide[tw] OR community health aides[tw] OR community health

nursing[tw] OR community medicine[tw] OR community health nurses[tw] OR community health nurse[tw] OR community health officers[tw] OR community health volunteer[tw] OR community health volunteers[tw] OR community health worker[tw] OR community health workers[tw] OR community IMCI[tw] OR community-oriented primary care[tw] OR community oriented primary care[tw] OR community volunteer[tw] OR community volunteers[tw] OR health extension workers[tw] OR integrated child development services[tw] OR Paramedical worker[tw] OR paramedical workers[tw] OR village development committee[tw] OR village development committees[tw] OR village health worker[tw] OR village health workers[tw] OR village health volunteer[tw] OR village health volunteers[tw] OR community-based nutrition programs[tw] OR community based nutrition programs[tw] OR community-based program[tw] OR community-based programs[tw] OR community-based programme[tw] OR community-based programmes[tw] OR community based programme[tw] OR community based programmes[tw] OR community involvement[tw] OR community participation[tw] OR community program[tw] OR community programs[tw] OR community programme[tw] OR community programmes[tw])

AND

("emerging country"[All Fields] OR "emerging countries"[All Fields] OR "emerging nation"[All Fields] OR "emerging nations"[All Fields] OR "emerging population"[All Fields] OR "emerging populations"[All Fields] OR "developing country"[Title/Abstract] OR "developing countries"[Title/Abstract] OR "developing nation"[Title/Abstract] OR "developing nations"[Title/Abstract] OR "developing population"[Title/Abstract] OR "developing populations"[Title/Abstract] OR "developing world"[Title/Abstract] OR "less developed country"[Title/Abstract] OR "less developed countries"[Title/Abstract] OR "less developed nation"[Title/Abstract] OR "less developed nations"[Title/Abstract] OR "less developed world"[Title/Abstract] OR "lesser developed countries"[Title/Abstract] OR "lesser developed nations"[Title/Abstract] OR "under developed country"[Title/Abstract] OR "under developed countries"[Title/Abstract] OR "under developed nations"[Title/Abstract] OR "under developed world"[Title/Abstract] OR "underdeveloped country"[Title/Abstract] OR "underdeveloped countries"[Title/Abstract] OR "underdeveloped nation"[Title/Abstract] OR "underdeveloped nations"[Title/Abstract] OR "underdeveloped population"[Title/Abstract] OR "underdeveloped populations"[Title/Abstract] OR "underdeveloped world"[Title/Abstract] OR "middle income country"[Title/Abstract] OR "middle income countries"[Title/Abstract] OR "middle income nation"[Title/Abstract] OR "middle income nations"[Title/Abstract] OR "middle income population"[Title/Abstract] OR "middle income populations"[Title/Abstract] OR "low income country"[Title/Abstract] OR "low income countries"[Title/Abstract] OR "low income nation"[Title/Abstract] OR "low income nations"[Title/Abstract] OR "low income population"[Title/Abstract] OR "low income populations"[Title/Abstract] OR "lower income country"[Title/Abstract] OR "lower income countries"[Title/Abstract] OR "lower income nations"[Title/Abstract] OR "lower income population"[Title/Abstract] OR "lower income populations"[Title/Abstract] OR "underserved countries"[Title/Abstract] OR "underserved nations"[Title/Abstract] OR "underserved population"[Title/Abstract] OR "underserved populations"[Title/Abstract] OR "under served population"[Title/Abstract] OR "under served populations"[Title/Abstract] OR "deprived countries"[Title/Abstract] OR "deprived population"[Title/Abstract] OR "deprived populations"[Title/Abstract] OR "poor country"[Title/Abstract] OR "poor countries"[Title/Abstract] OR

"poor nation"[Title/Abstract] OR "poor nations"[Title/Abstract] OR "poor population"[Title/Abstract] OR "poor populations"[Title/Abstract] OR "poor world"[Title/Abstract] OR "poorer countries"[Title/Abstract] OR "poorer nations"[Title/Abstract] OR "poorer population"[Title/Abstract] OR "poorer populations"[Title/Abstract] OR "developing economy"[Title/Abstract] OR "developing economies"[Title/Abstract] OR "less developed economy"[Title/Abstract] OR "less developed economies"[Title/Abstract] OR "underdeveloped economies"[Title/Abstract] OR "middle income economy"[Title/Abstract] OR "middle income economies"[Title/Abstract] OR "low income economy"[Title/Abstract] OR "low income economies"[Title/Abstract] OR "lower income economies"[Title/Abstract] OR "low gdp"[Title/Abstract] OR "low gnp"[Title/Abstract] OR "low gross domestic"[Title/Abstract] OR "low gross national"[Title/Abstract] OR "lower gdp"[Title/Abstract] OR "lower gross domestic"[Title/Abstract] OR "lmic"[Title/Abstract] OR "lmics"[Title/Abstract] OR "third world"[Title/Abstract] OR "lami country"[Title/Abstract] OR "lami countries"[Title/Abstract] OR "transitional country"[Title/Abstract] OR "transitional countries"[Title/Abstract] OR "Africa"[Title/Abstract] OR "Asia"[Title/Abstract] OR "Caribbean"[Title/Abstract] OR "west indies"[Title/Abstract] OR "south america"[Title/Abstract] OR "latin america"[Title/Abstract] OR "central america"[Title/Abstract] OR "Atlantic Islands"[Title/Abstract] OR "Commonwealth of Independent States"[Title/Abstract] OR "Pacific Islands"[Title/Abstract] OR "Indian Ocean Islands"[Title/Abstract] OR "Eastern Europe"[Title/Abstract] OR "Afghanistan"[Title/Abstract] OR "Albania"[Title/Abstract] OR "Algeria"[Title/Abstract] OR "Angola"[Title/Abstract] OR "Armenia"[Title/Abstract] OR "Armenian"[Title/Abstract] OR "Azerbaijan"[Title/Abstract] OR "Bangladesh"[Title/Abstract] OR "Benin"[Title/Abstract] OR "Byelarus"[Title/Abstract] OR "Byelorussian"[Title/Abstract] OR "Belarus"[Title/Abstract] OR "Belorussian"[Title/Abstract] OR "Belorussia"[Title/Abstract] OR "Belize"[Title/Abstract] OR "Bhutan"[Title/Abstract] OR "Bolivia"[Title/Abstract] OR "Bosnia"[Title/Abstract] OR "Herzegovina"[Title/Abstract] OR "Hercegovina"[Title/Abstract] OR "Botswana"[Title/Abstract] OR "Brasil"[Title/Abstract] OR "Brazil"[Title/Abstract] OR "Bulgaria"[Title/Abstract] OR "burkina faso"[Title/Abstract] OR "burkina fasso"[Title/Abstract] OR "upper volta"[Title/Abstract] OR "Burundi"[Title/Abstract] OR "Urundi"[Title/Abstract] OR "Cambodia"[Title/Abstract] OR "khmer republic"[Title/Abstract] OR "Kampuchea"[Title/Abstract] OR "Cameroon"[Title/Abstract] OR "Cameroons"[Title/Abstract] OR "Cameron"[Title/Abstract] OR "cape verde"[Title/Abstract] OR "central african republic"[Title/Abstract] OR "Chad"[Title/Abstract] OR "China"[Title/Abstract] OR "Taiwan"[Title/Abstract] OR "Taiwan"[MeSH Terms] OR "Colombia"[Title/Abstract] OR "Comoros"[Title/Abstract] OR "comoro islands"[Title/Abstract] OR "Comores"[Title/Abstract] OR "Mayotte"[Title/Abstract] OR "Congo"[Title/Abstract] OR "Zaire"[Title/Abstract] OR "costa rica"[Title/Abstract] OR "cote d'ivoire"[Title/Abstract] OR "ivory coast"[Title/Abstract] OR "Cuba"[Title/Abstract] OR "Czechoslovakia"[Title/Abstract] OR "Slovakia"[Title/Abstract] OR "Djibouti"[Title/Abstract] OR "french somaliland"[Title/Abstract] OR "Dominica"[Title/Abstract] OR "dominican republic"[Title/Abstract] OR "east timor"[Title/Abstract] OR "East Timur"[Title/Abstract] OR "timor-leste"[Title/Abstract] OR "Ecuador"[Title/Abstract] OR "Egypt"[Title/Abstract] OR "el salvador"[Title/Abstract] OR "Eritrea"[Title/Abstract] OR "Ethiopia"[Title/Abstract] OR "Fiji"[Title/Abstract] OR "Gabon"[Title/Abstract] OR "gabonese republic"[Title/Abstract] OR "Gambia"[Title/Abstract] OR "Gaza"[Title/Abstract] OR "georgia republic"[Title/Abstract] OR "georgian republic"[Title/Abstract] OR "Ghana"[Title/Abstract] OR "gold coast"[Title/Abstract] OR "Grenada"[Title/Abstract] OR "Guatemala"[Title/Abstract] OR "Guinea"[Title/Abstract] OR "Guiana"[Title/Abstract] OR "Guyana"[Title/Abstract] OR

"Haiti"[Title/Abstract] OR "Honduras"[Title/Abstract] OR "India"[Title/Abstract] OR "Maldives"[Title/Abstract] OR "Indonesia"[Title/Abstract] OR "Iran"[Title/Abstract] OR "Iraq"[Title/Abstract] OR "Jamaica"[Title/Abstract] OR "Jordan"[Title/Abstract] OR "Kazakhstan"[Title/Abstract] OR "Kazakh"[Title/Abstract] OR "Kenya"[Title/Abstract] OR "Kiribati"[Title/Abstract] OR "Korea"[Title/Abstract] OR "Kosovo"[Title/Abstract] OR "Kyrgyzstan"[Title/Abstract] OR "Kirghizia"[Title/Abstract] OR "kyrgyz republic"[Title/Abstract] OR "Kirghiz"[Title/Abstract] OR "Kirgizstan"[Title/Abstract] OR "Lao PDR"[Title/Abstract] OR "Laos"[Title/Abstract] OR "Lebanon"[Title/Abstract] OR "Lesotho"[Title/Abstract] OR "Basutoland"[Title/Abstract] OR "Liberia"[Title/Abstract] OR "Libya"[Title/Abstract] OR "Macedonia"[Title/Abstract] OR "Madagascar"[Title/Abstract] OR "malagasy republic"[Title/Abstract] OR "Malaysia"[Title/Abstract] OR "Malaya"[Title/Abstract] OR "Malay"[Title/Abstract] OR "Sabah"[Title/Abstract] OR "Sarawak"[Title/Abstract] OR "Malawi"[Title/Abstract] OR "Nyasaland"[Title/Abstract] OR "Mali"[Title/Abstract] OR "marshall islands"[Title/Abstract] OR "Mauritania"[Title/Abstract] OR "Mauritius"[Title/Abstract] OR "agalega islands"[Title/Abstract] OR "Melanesia"[Title/Abstract] OR "Mexico"[Title/Abstract] OR "Micronesia"[Title/Abstract] OR "middle east"[Title/Abstract] OR "Moldova"[Title/Abstract] OR "Moldovia"[Title/Abstract] OR "Moldovian"[Title/Abstract] OR "Mongolia"[Title/Abstract] OR "Montenegro"[Title/Abstract] OR "Morocco"[Title/Abstract] OR "Ifni"[Title/Abstract] OR "Mozambique"[Title/Abstract] OR "Myanmar"[Title/Abstract] OR "Myanma"[Title/Abstract] OR "Burma"[Title/Abstract] OR "Namibia"[Title/Abstract] OR "Nepal"[Title/Abstract] OR "Nicaragua"[Title/Abstract] OR "Niger"[Title/Abstract] OR "Nigeria"[Title/Abstract] OR "Muscat"[Title/Abstract] OR "Pakistan"[Title/Abstract] OR "Palau"[Title/Abstract] OR "Palestine"[Title/Abstract] OR "Panama"[Title/Abstract] OR "Paraguay"[Title/Abstract] OR "Peru"[Title/Abstract] OR "Philippines"[Title/Abstract] OR "Philipines"[Title/Abstract] OR "Phillipines"[Title/Abstract] OR "Phillippines"[Title/Abstract] OR "Romania"[Title/Abstract] OR "Rumania"[Title/Abstract] OR "Roumania"[Title/Abstract] OR "Rwanda"[Title/Abstract] OR "Ruanda"[Title/Abstract] OR "saint kitts"[Title/Abstract] OR "st kitts"[Title/Abstract] OR "Nevis"[Title/Abstract] OR "saint lucia"[Title/Abstract] OR "st lucia"[Title/Abstract] OR "saint vincent"[Title/Abstract] OR "st vincent"[Title/Abstract] OR "Grenadines"[Title/Abstract] OR "Samoa"[Title/Abstract] OR "samoan islands"[Title/Abstract] OR "Navigator Island" [Title/Abstract] OR "Navigator Islands" [Title/Abstract] OR "sao tome"[Title/Abstract] OR "Senegal"[Title/Abstract] OR "Serbia"[Title/Abstract] OR "Montenegro"[Title/Abstract] OR "sierra leone"[Title/Abstract] OR "sri lanka"[Title/Abstract] OR "Ceylon"[Title/Abstract] OR "solomon islands"[Title/Abstract] OR "Somalia"[Title/Abstract] OR "Sudan"[Title/Abstract] OR "Suriname"[Title/Abstract] OR "Surinam"[Title/Abstract] OR "Swaziland"[Title/Abstract] OR "Syria"[Title/Abstract] OR "Syrian"[Title/Abstract] OR "Tajikistan"[Title/Abstract] OR "Tadzhikistan"[Title/Abstract] OR "Tadjikistan"[Title/Abstract] OR "Tadzhik"[Title/Abstract] OR "Tanzania"[Title/Abstract] OR "Thailand"[Title/Abstract] OR "Togo"[Title/Abstract] OR "togolese republic"[Title/Abstract] OR "Tonga"[Title/Abstract] OR "Tunisia"[Title/Abstract] OR "Turkey"[Title/Abstract] OR "Turkmenistan"[Title/Abstract] OR "Turkmen"[Title/Abstract] OR "Tuvalu"[Title/Abstract] OR "Uganda"[Title/Abstract] OR "Ukraine"[Title/Abstract] OR "Uzbekistan"[Title/Abstract] OR "uzbek"[Title/Abstract] OR "Vanuatu"[Title/Abstract] OR "new hebrides"[Title/Abstract] OR "Vietnam"[Title/Abstract] OR "viet nam"[Title/Abstract] OR "west bank"[Title/Abstract] OR "Yemen"[Title/Abstract] OR "Yugoslavia"[Title/Abstract] OR "Zambia"[Title/Abstract] OR "Zimbabwe"[Title/Abstract] OR

"Rhodesia"[Title/Abstract] OR "developing countries"[MeSH Terms] OR "Africa"[MeSH Terms:noexp] OR "africa, northern"[MeSH Terms:noexp] OR "africa south of the sahara"[MeSH Terms:noexp] OR "africa, central"[MeSH Terms:noexp] OR "africa, eastern"[MeSH Terms:noexp] OR "africa, southern"[MeSH Terms:noexp] OR "africa, western"[MeSH Terms:noexp] OR "Asia"[MeSH Terms:noexp] OR "asia, central"[MeSH Terms:noexp] OR "asia, southeastern"[MeSH Terms:noexp] OR "asia, western"[MeSH Terms:noexp] OR "caribbean region"[MeSH Terms:noexp] OR "west indies"[MeSH Terms:noexp] OR "south america"[MeSH Terms:noexp] OR "latin america"[MeSH Terms:noexp] OR "central america"[MeSH Terms:noexp] OR "Atlantic Islands"[MeSH Terms:noexp] OR "Commonwealth of Independent States"[MeSH Terms:noexp] OR "Pacific Islands"[MeSH Terms:noexp] OR "Indian Ocean Islands"[MeSH Terms:noexp] OR "europe, eastern"[MeSH Terms:noexp] OR "Afghanistan"[MeSH Terms] OR "Albania"[MeSH Terms] OR "Algeria"[MeSH Terms] OR "american samoa"[MeSH Terms] OR "Angola"[MeSH Terms] OR "Armenia"[MeSH Terms] OR "Azerbaijan"[MeSH Terms] OR "Baltic States"[MeSH Terms] OR "Bangladesh"[MeSH Terms] OR "Benin"[MeSH Terms] OR "Republic of Belarus"[MeSH Terms] OR "Belize"[MeSH Terms] OR "Bhutan"[MeSH Terms] OR "Bolivia"[MeSH Terms] OR "bosnia and herzegovina"[MeSH Terms] OR "Botswana"[MeSH Terms] OR "Brazil"[MeSH Terms] OR "Bulgaria"[MeSH Terms] OR "burkina faso"[MeSH Terms] OR "Burundi"[MeSH Terms] OR "Cambodia"[MeSH Terms] OR "Cameroon"[MeSH Terms] OR "cabo verde"[MeSH Terms] OR "central african republic"[MeSH Terms] OR "Chad"[MeSH Terms] OR "China"[MeSH Terms] OR "Colombia"[MeSH Terms] OR "Comoros"[MeSH Terms] OR "Congo"[MeSH Terms] OR "costa rica"[MeSH Terms] OR "cote d'ivoire"[MeSH Terms] OR "Cuba"[MeSH Terms] OR "Czechoslovakia"[MeSH Terms] OR "Slovakia"[MeSH Terms] OR "Djibouti"[MeSH Terms] OR "Democratic Republic of the Congo"[MeSH Terms] OR "Democratic People's Republic of Korea"[MeSH Terms] OR "Dominica"[MeSH Terms] OR "dominican republic"[MeSH Terms] OR "timor-leste"[MeSH Terms] OR "Ecuador"[MeSH Terms] OR "Egypt"[MeSH Terms] OR "el salvador"[MeSH Terms] OR "Eritrea"[MeSH Terms] OR "Ethiopia"[MeSH Terms] OR "Fiji"[MeSH Terms] OR "French Guiana"[MeSH Terms] OR "Gabon"[MeSH Terms] OR "Gambia"[MeSH Terms] OR "georgia (republic)"[MeSH Terms] OR "Ghana"[MeSH Terms] OR "Grenada"[MeSH Terms] OR "Guatemala"[MeSH Terms] OR "Guinea"[MeSH Terms] OR "guinea-bissau"[MeSH Terms] OR "Guyana"[MeSH Terms] OR "Haiti"[MeSH Terms] OR "Honduras"[MeSH Terms] OR "Independent State of Samoa"[MeSH Terms] OR "India"[MeSH Terms] OR "Indonesia"[MeSH Terms] OR "Iran"[MeSH Terms] OR "Iraq"[MeSH Terms] OR "Jamaica"[MeSH Terms] OR "Jordan"[MeSH Terms] OR "Kazakhstan"[MeSH Terms] OR "Kenya"[MeSH Terms] OR "Korea"[MeSH Terms] OR "Kyrgyzstan"[MeSH Terms] OR "Laos"[MeSH Terms] OR "Lebanon"[MeSH Terms] OR "Lesotho"[MeSH Terms] OR "Liberia"[MeSH Terms] OR "Libya"[MeSH Terms] OR "Madagascar"[MeSH Terms] OR "Malawi"[MeSH Terms] OR "Malaysia"[MeSH Terms] OR "Mali"[MeSH Terms] OR "Mauritania"[MeSH Terms] OR "Mauritius"[MeSH Terms] OR "Melanesia"[MeSH Terms] OR "Mexico"[MeSH Terms] OR "Micronesia"[MeSH Terms] OR "middle east"[MeSH Terms:noexp] OR "Moldova"[MeSH Terms] OR "Mongolia"[MeSH Terms] OR "Montenegro"[MeSH Terms] OR "Morocco"[MeSH Terms] OR "Mozambique"[MeSH Terms] OR "Myanmar"[MeSH Terms] OR "Namibia"[MeSH Terms] OR "Nepal"[MeSH Terms] OR "Nicaragua"[MeSH Terms] OR "Niger"[MeSH Terms] OR "Nigeria"[MeSH Terms] OR "Pakistan"[MeSH Terms] OR "Palau"[MeSH Terms] OR "Panama"[MeSH Terms] OR "papua new guinea"[MeSH Terms] OR "Paraguay"[MeSH Terms] OR "Peru"[MeSH Terms] OR "Philippines"[MeSH Terms] OR "Republic of Korea"[MeSH Terms] OR "Republic of North Macedonia"[MeSH Terms] OR "Romania"[MeSH Terms] OR "Rwanda"[MeSH Terms] OR "saint lucia"[MeSH Terms] OR "Saint Vincent and the Grenadines"[MeSH Terms] OR "Samoa"[MeSH Terms] OR "Senegal"[MeSH Terms] OR "Serbia"[MeSH Terms] OR

"Montenegro"[MeSH Terms] OR "sierra leone"[MeSH Terms] OR "sri lanka"[MeSH Terms] OR "Somalia"[MeSH Terms] OR "south africa"[MeSH Terms] OR "Sudan"[MeSH Terms] OR "Suriname"[MeSH Terms] OR "eswatini"[MeSH Terms] OR "Syria"[MeSH Terms] OR "Tajikistan"[MeSH Terms] OR "Tanzania"[MeSH Terms] OR "Thailand"[MeSH Terms] OR "Togo"[MeSH Terms] OR "Tonga"[MeSH Terms] OR "Tunisia"[MeSH Terms] OR "Turkey"[MeSH Terms] OR "Turkmenistan"[MeSH Terms] OR "Uganda"[MeSH Terms] OR "Ukraine"[MeSH Terms] OR "Uzbekistan"[MeSH Terms] OR "Vanuatu"[MeSH Terms] OR "Vietnam"[MeSH Terms] OR "Yemen"[MeSH Terms] OR "Yugoslavia"[MeSH Terms] OR "Zambia"[MeSH Terms] OR "Zimbabwe"[MeSH Terms] OR "Southern African Development Community"[All Fields] OR "East African Community"[All Fields] OR "West African Health Organisation"[All Fields] OR "Sub Saharan Africa"[All Fields] OR "SubSaharan Africa"[All Fields])

EMBASE

'Human immunodeficiency virus'/exp OR 'Human immunodeficiency virus 1'/exp OR 'Human immunodeficiency virus 2'/exp OR 'acquired immune deficiency syndrome'/exp OR 'hiv infections transmission'/exp OR ("HIV" OR "AIDS" OR "Human immunodeficiency viruses" OR "HTLV-III" OR "Human Immunodeficiency Virus" OR "Acquired Immune Deficiency" OR "Acquired Immunodeficiency" OR "T Lymphotropic Virus Type III Infections" OR "T-Lymphotropic Virus Type III Infection" OR "acquired immunologic deficiency"):ab,ti

AND

'community medicine'/exp OR 'community health nursing'/exp OR 'health auxiliary'/exp OR 'child welfare'/exp OR ("maternal self-administration" OR "child welfare" OR "Community health workers" OR "Community Networks" OR "Community Health Aides" OR "Community Health Nursing" OR "Community Medicine" OR "community network" OR "community networks" OR "community health aide" OR "community health aides" OR "community health nursing" OR "community medicine" OR "community health nurses" OR "community health nurse" OR "community health officers" OR "community health volunteer" OR "community health volunteers" OR "community health worker" OR "community health workers" OR "community IMCI" OR "community-oriented primary care" OR "community oriented primary care" OR "community volunteer" OR "community volunteers" OR "health extension workers" OR "integrated child development services" OR "Paramedical worker" OR "paramedical workers" OR "village development committee" OR "village development committees" OR "village health worker" OR "village health workers" OR "village health volunteer" OR "village health volunteers" OR "community-based nutrition programs" OR "community based nutrition programs" OR "community-based program" OR "community-based programs" OR "community-based programme" OR "community-based programmes" OR "community based programme" OR "community based programmes" OR "community involvement" OR "community participation" OR "community program" OR "community programs" OR "community programme" OR "community programmes")

AND

'developing country':ab,ti OR 'developing countries':ab,ti OR 'developing nation':ab,ti OR 'developing nations':ab,ti OR 'developing population':ab,ti OR 'developing populations':ab,ti OR 'developing world':ab,ti OR 'less developed country':ab,ti OR 'less developed countries':ab,ti OR 'less developed nation':ab,ti OR 'less developed nations':ab,ti OR 'less developed population':ab,ti OR 'less developed populations':ab,ti OR 'less developed world':ab,ti OR

'lesser developed country':ab,ti OR 'lesser developed countries':ab,ti OR 'lesser developed nation':ab,ti OR 'lesser developed nations':ab,ti OR 'lesser developed population':ab,ti OR 'lesser developed populations':ab,ti OR 'lesser developed world':ab,ti OR 'under developed country':ab,ti OR 'under developed countries':ab,ti OR 'under developed nation':ab,ti OR 'under developed nations':ab,ti OR 'under developed population':ab,ti OR 'under developed populations':ab,ti OR 'under developed world':ab,ti OR 'underdeveloped country':ab,ti OR 'underdeveloped countries':ab,ti OR 'underdeveloped nation':ab,ti OR 'underdeveloped nations':ab,ti OR 'underdeveloped population':ab,ti OR 'underdeveloped populations':ab,ti OR 'underdeveloped world':ab,ti OR 'middle income country':ab,ti OR 'middle income countries':ab,ti OR 'middle income nation':ab,ti OR 'middle income nations':ab,ti OR 'middle income population':ab,ti OR 'middle income populations':ab,ti OR 'low income country':ab,ti OR 'low income countries':ab,ti OR 'low income nation':ab,ti OR 'low income nations':ab,ti OR 'low income population':ab,ti OR 'low income populations':ab,ti OR 'lower income country':ab,ti OR 'lower income countries':ab,ti OR 'lower income nation':ab,ti OR 'lower income nations':ab,ti OR 'lower income population':ab,ti OR 'lower income populations':ab,ti OR 'underserved country':ab,ti OR 'underserved countries':ab,ti OR 'underserved nation':ab,ti OR 'underserved nations':ab,ti OR 'underserved population':ab,ti OR 'underserved populations':ab,ti OR 'underserved world':ab,ti OR 'under served country':ab,ti OR 'under served countries':ab,ti OR 'under served nation':ab,ti OR 'under served nations':ab,ti OR 'under served population':ab,ti OR 'under served populations':ab,ti OR 'under served world':ab,ti OR 'deprived country':ab,ti OR 'deprived countries':ab,ti OR 'deprived nation':ab,ti OR 'deprived nations':ab,ti OR 'deprived population':ab,ti OR 'deprived populations':ab,ti OR 'deprived world':ab,ti OR 'poor country':ab,ti OR 'poor countries':ab,ti OR 'poor nation':ab,ti OR 'poor nations':ab,ti OR 'poor population':ab,ti OR 'poor populations':ab,ti OR 'poor world':ab,ti OR 'poorer country':ab,ti OR 'poorer countries':ab,ti OR 'poorer nation':ab,ti OR 'poorer nations':ab,ti OR 'poorer population':ab,ti OR 'poorer populations':ab,ti OR 'poorer world':ab,ti OR 'developing economy':ab,ti OR 'developing economies':ab,ti OR 'less developed economy':ab,ti OR 'less developed economies':ab,ti OR 'lesser developed economy':ab,ti OR 'lesser developed economies':ab,ti OR 'under developed economy':ab,ti OR 'under developed economies':ab,ti OR 'underdeveloped economy':ab,ti OR 'underdeveloped economies':ab,ti OR 'middle income economy':ab,ti OR 'middle income economies':ab,ti OR 'low income economy':ab,ti OR 'low income economies':ab,ti OR 'lower income economy':ab,ti OR 'lower income economies':ab,ti OR 'low gdp':ab,ti OR 'low gnp':ab,ti OR 'low gross domestic':ab,ti OR 'low gross national':ab,ti OR 'lower gdp':ab,ti OR 'lower gnp':ab,ti OR 'lower gross domestic':ab,ti OR 'lower gross national':ab,ti OR lmic:ab,ti OR lmics:ab,ti OR 'third world':ab,ti OR 'lami country':ab,ti OR 'lami countries':ab,ti OR 'transitional country':ab,ti OR 'transitional countries':ab,ti OR Africa:ti,ab OR Asia:ti,ab OR Caribbean:ti,ab OR 'West Indies':ti,ab OR 'South America':ti,ab OR 'Latin America':ti,ab OR 'Central America':ti,ab OR 'atlantic islands':ab,ti OR 'commonwealth of independent states':ab,ti OR 'pacific islands':ab,ti OR 'indian ocean islands':ab,ti OR 'eastern europe':ab,ti OR Afghanistan:ti,ab OR Albania:ti,ab OR Algeria:ti,ab OR Angola:ti,ab OR Antigua:ti,ab OR Barbuda:ti,ab OR Argentina:ti,ab OR Armenia:ti,ab OR Armenian:ti,ab OR Aruba:ti,ab OR Azerbaijan:ti,ab OR Bahrain:ti,ab OR Bangladesh:ti,ab OR Barbados:ti,ab OR

Benin:ti,ab OR Byelarus:ti,ab OR Byelorussian:ti,ab OR Belarus:ti,ab OR Belorussian:ti,ab OR Belorussia:ti,ab OR Belize:ti,ab OR Bhutan:ti,ab OR Bolivia:ti,ab OR Bosnia:ti,ab OR Herzegovina:ti,ab OR Hercegovina:ti,ab OR Botswana:ti,ab OR Brasil:ti,ab OR Brazil:ti,ab OR Bulgaria:ti,ab OR 'Burkina Faso':ti,ab OR 'Burkina Fasso':ti,ab OR 'Upper Volta':ti,ab OR Burundi:ti,ab OR Urundi:ti,ab OR Cambodia:ti,ab OR 'Khmer Republic':ti,ab OR Kampuchea:ti,ab OR Cameroon:ti,ab OR Cameroons:ti,ab OR Cameron:ti,ab OR Camerons:ti,ab OR 'Cape Verde':ti,ab OR 'Central African Republic':ti,ab OR Chad:ti,ab OR Chile:ti,ab OR China:ti,ab OR Colombia:ti,ab OR Comoros:ti,ab OR 'Comoro Islands':ti,ab OR Comores:ti,ab OR Mayotte:ti,ab OR Congo:ti,ab OR Zaire:ti,ab OR 'Costa Rica':ti,ab OR 'Cote d`Ivoire' OR 'Ivory Coast':ti,ab OR Croatia:ti,ab OR Cuba:ti,ab OR Cyprus:ti,ab OR Czechoslovakia:ti,ab OR 'Czech Republic':ti,ab OR Slovakia:ti,ab OR 'Slovak Republic':ti,ab OR Djibouti:ti,ab OR 'French Somaliland':ti,ab OR Dominica:ti,ab OR 'Dominican Republic':ti,ab OR 'East Timor':ti,ab OR 'East Timur':ti,ab OR 'Timor Leste':ti,ab OR Ecuador:ti,ab OR Egypt:ti,ab OR 'United Arab Republic':ti,ab OR El Salvador:ti,ab OR Eritrea:ti,ab OR Estonia:ti,ab OR Ethiopia:ti,ab OR Fiji:ti,ab OR Gabon:ti,ab OR 'Gabonese Republic':ti,ab OR Gambia:ti,ab OR Gaza:ti,ab OR 'Georgia Republic':ti,ab OR 'Georgian Republic':ti,ab OR Ghana:ti,ab OR 'Gold Coast':ti,ab OR Greece:ti,ab OR Grenada:ti,ab OR Guatemala:ti,ab OR Guinea:ti,ab OR Guam:ti,ab OR Guiana:ti,ab OR Guyana:ti,ab OR Haiti:ti,ab OR Honduras:ti,ab OR Hungary:ti,ab OR India:ti,ab OR Maldives:ti,ab OR Indonesia:ti,ab OR Iran:ti,ab OR Iraq:ti,ab OR 'Isle of Man':ti,ab OR Jamaica:ti,ab OR Jordan:ti,ab OR Kazakhstan:ti,ab OR Kazakh:ti,ab OR Kenya:ti,ab OR Kiribati:ti,ab OR Korea:ti,ab OR Kosovo:ti,ab OR Kyrgyzstan:ti,ab OR Kirghizia:ti,ab OR 'Kyrgyz Republic':ti,ab OR Kirghiz:ti,ab OR Kirgizstan:ti,ab OR 'Lao PDR':ti,ab OR Laos:ti,ab OR Latvia:ti,ab OR Lebanon:ti,ab OR Lesotho:ti,ab OR Basutoland:ti,ab OR Liberia:ti,ab OR Libya:ti,ab OR Lithuania:ti,ab OR Macedonia:ti,ab OR Madagascar:ti,ab OR 'Malagasy Republic':ti,ab OR Malaysia:ti,ab OR Malaya:ti,ab OR Malay:ti,ab OR Sabah:ti,ab OR Sarawak:ti,ab OR Malawi:ti,ab OR Nyasaland:ti,ab OR Mali:ti,ab OR Malta:ti,ab OR 'Marshall Islands':ti,ab OR Mauritania:ti,ab OR Mauritius:ti,ab OR melanesia:ab,ti OR 'Agalega Islands':ti,ab OR Mexico:ti,ab OR Micronesia:ti,ab OR 'Middle East':ti,ab OR Moldova:ti,ab OR Moldovia:ti,ab OR Moldovian:ti,ab OR Mongolia:ti,ab OR Montenegro:ti,ab OR Morocco:ti,ab OR Ifni:ti,ab OR Mozambique:ti,ab OR Myanmar:ti,ab OR Myanma:ti,ab OR Burma:ti,ab OR Namibia:ti,ab OR Nepal:ti,ab OR 'Netherlands Antilles':ti,ab OR 'New Caledonia':ti,ab OR Nicaragua:ti,ab OR Niger:ti,ab OR Nigeria:ti,ab OR 'Northern Mariana Islands':ti,ab OR Oman:ti,ab OR Muscat:ti,ab OR Pakistan:ti,ab OR Palau:ti,ab OR Palestine:ti,ab OR Panama:ti,ab OR Paraguay:ti,ab OR Peru:ti,ab OR Philippines:ti,ab OR Philipines:ti,ab OR Phillipines:ti,ab OR Phillippines:ti,ab OR Poland:ti,ab OR Portugal:ti,ab OR 'Puerto Rico':ti,ab OR Romania:ti,ab OR Rumania:ti,ab OR Roumania:ti,ab OR Russia:ti,ab OR Russian:ti,ab OR Rwanda:ti,ab OR Ruanda:ti,ab OR 'Saint Kitts':ti,ab OR 'St Kitts':ti,ab OR Nevis:ti,ab OR 'Saint Lucia':ti,ab OR 'St Lucia':ti,ab OR 'Saint Vincent':ti,ab OR 'St Vincent':ti,ab OR Grenadines:ti,ab OR Samoa:ti,ab OR 'Samoan Islands':ti,ab OR 'Navigator Island':ti,ab OR 'Navigator Islands':ti,ab OR 'Sao Tome':ti,ab OR 'Saudi Arabia':ti,ab OR Senegal:ti,ab OR Serbia:ti,ab OR Montenegro:ti,ab OR Seychelles:ti,ab OR 'Sierra Leone':ti,ab OR Slovenia:ti,ab OR 'Sri Lanka':ti,ab OR Ceylon:ti,ab OR 'Solomon Islands':ti,ab OR Somalia:ti,ab OR Sudan:ti,ab OR Suriname:ti,ab OR Surinam:ti,ab OR Swaziland:ti,ab OR Syria:ti,ab OR Syrian:ti,ab OR Tajikistan:ti,ab OR

Tadzhikistan:ti,ab OR Tadjikistan:ti,ab OR Tadzhik:ti,ab OR Tanzania:ti,ab OR Thailand:ti,ab OR Togo:ti,ab OR 'Togolese Republic':ti,ab OR Tonga:ti,ab OR Trinidad:ti,ab OR Tobago:ti,ab OR Tunisia:ti,ab OR Turkey:ti,ab OR Turkmenistan:ti,ab OR Turkmen:ti,ab OR Tuvalu:ti,ab OR Uganda:ti,ab OR Ukraine:ti,ab OR Uruguay:ti,ab OR USSR:ti,ab OR 'Soviet Union':ti,ab OR 'Union of Soviet Socialist Republics':ti,ab OR Uzbekistan:ti,ab OR Uzbek OR Vanuatu:ti,ab OR 'New Hebrides':ti,ab OR Venezuela:ti,ab OR Vietnam:ti,ab OR 'Viet Nam':ti,ab OR 'West Bank':ti,ab OR Yemen:ti,ab OR Yugoslavia:ti,ab OR Zambia:ti,ab OR Zimbabwe:ti,ab OR Rhodesia:ti,ab OR 'developing country'/exp OR 'Africa'/de OR 'Africa south of the Sahara'/de OR 'North Africa'/de OR 'Central Africa'/de OR 'Asia'/de OR 'South Asia'/de OR 'Southeast Asia'/de OR 'South America'/de OR 'Central America'/de OR 'South and Central America'/de OR 'Atlantic islands'/de OR 'Caribbean Islands'/de OR 'Pacific islands'/de OR 'Indian Ocean'/de OR 'Eastern Europe'/de OR Afghanistan/exp OR Albania/exp OR Algeria/exp OR 'American Samoa'/exp OR Angola/exp OR 'Antigua and Barbuda'/exp OR Argentina/exp OR Armenia/exp OR Azerbaijan/exp OR Bahrain/exp OR Bangladesh/exp OR Barbados/exp OR Benin/exp OR 'Belarus'/exp OR 'Baltic States'/exp OR Belize/exp OR Bhutan/exp OR Bolivia/exp OR 'Bosnia and Herzegovina'/exp OR Botswana/exp OR Brazil/exp OR Bulgaria/exp OR 'Burkina Faso'/exp OR Burundi/exp OR Cambodia/exp OR Cameroon/exp OR 'Cape Verde'/exp OR 'Central African Republic'/exp OR Chad/exp OR Chile/exp OR China/exp OR Colombia/exp OR Comoros/exp OR Congo/exp OR 'Costa Rica'/exp OR 'Cote d`Ivoire'/exp OR Croatia/exp OR Cuba/exp OR Cyprus/exp OR Czechoslovakia/exp OR 'Czech Republic'/exp OR Slovakia/exp OR Djibouti/exp OR 'Democratic Republic Congo'/exp OR Dominica/exp OR 'Dominican Republic'/exp OR 'Timor-Leste'/exp OR Ecuador/exp OR Egypt/exp OR 'El Salvador'/exp OR Eritrea/exp OR Estonia/exp OR Ethiopia/exp OR 'French Guiana'/exp OR Fiji/exp OR Gabon/exp OR Gambia/exp OR 'Georgia (Republic) '/exp OR Ghana/exp OR Greece/exp OR Grenada/exp OR Guatemala/exp OR Guinea/exp OR Guinea-Bissau/exp OR Guam/exp OR Guyana/exp OR Haiti/exp OR Honduras/exp OR Hungary/exp OR India/exp OR Indonesia/exp OR Iran/exp OR Iraq/exp OR Jamaica/exp OR Jordan/exp OR Kazakhstan/exp OR Kenya/exp OR Korea/exp OR Kyrgyzstan/exp OR Laos/exp OR Latvia/exp OR Lebanon/exp OR Lesotho/exp OR Liberia/exp OR 'Libyan Arab Jamahiriya'/exp OR Lithuania/exp OR 'Macedonia (republic)'/exp OR Madagascar/exp OR Malaysia/exp OR Malawi/exp OR Mali/exp OR Malta/exp OR Mauritania/exp OR Mauritius/exp OR "Melanesia"/exp **OR** Mexico/exp OR 'Federated States of Micronesia'/exp OR 'Middle East'/de OR Moldova/exp OR Mongolia/exp OR Montenegro/exp OR Morocco/exp OR Mozambique/exp OR Myanmar/exp OR Namibia/exp OR Nepal/exp OR 'Netherlands Antilles'/exp OR 'New Caledonia'/exp OR Nicaragua/exp OR Niger/exp OR Nigeria/exp OR 'North Korea'/exp OR Oman/exp OR Pakistan/exp OR Palau/exp OR Panama/exp OR 'Papua New Guinea'/exp OR Paraguay/exp OR Peru/exp OR Philippines/exp OR Poland/exp OR Portugal/exp OR 'Puerto Rico'/exp OR Romania/exp OR 'Russian Federation'/exp OR Rwanda/exp OR 'Saint Kitts and Nevis'/exp OR 'Saint Lucia'/exp OR 'Saint Vincent and the Grenadines'/exp OR 'Samoan Islands'/exp OR Samoa/exp OR 'Saudi Arabia'/exp OR Senegal/exp OR Serbia/exp OR 'Montenegro (republic)'/exp OR

Seychelles/exp OR 'Sierra Leone'/exp OR Slovenia/exp OR 'Sri Lanka'/exp OR Somalia/exp OR 'South Korea'/exp OR 'South Africa'/exp OR Sudan/exp OR Suriname/exp OR Swaziland/exp OR 'Syrian Arab Republic'/exp OR Tajikistan/exp OR Tanzania/exp OR Thailand/exp OR Togo/exp OR Tonga/exp OR 'Trinidad and Tobago'/exp OR Tunisia/exp OR 'Turkey (republic)'/exp OR Turkmenistan/exp OR Uganda/exp OR Ukraine/exp OR Uruguay/exp OR USSR/exp OR Uzbekistan/exp OR Vanuatu/exp OR Venezuela/exp OR 'Viet Nam'/exp OR Yemen/exp OR Yugoslavia/exp OR 'Yugoslavia (pre-1992)'/exp OR Zambia/exp OR Zimbabwe/exp

Ovid Global Health Database

(Exp Human immunodeficiency viruses/ OR exp Human immunodeficiency virus 1/ OR exp Human immunodeficiency virus 2/ OR exp acquired immune deficiency syndrome/ OR exp hiv infections/) OR ("HIV" OR "AIDS" OR "Human immunodeficiency viruses" OR "HTLV-III" OR "Human Immunodeficiency Virus" OR "Acquired Immune Deficiency" OR "Acquired Immunodeficiency" OR "T Lymphotropic Virus Type III Infections" OR "T-Lymphotropic Virus Type III Infection" OR "acquired immunologic deficiency")

AND
Exp child welfare/ OR ("maternal self-administration" OR "child welfare" OR "Community health workers" OR "Community Networks" OR "Community Health Aides" OR "Community Health Nursing" OR "Community Medicine" OR "community network" OR "community networks" OR "community health aide" OR "community health aides" OR "community health nursing" OR "community medicine" OR "community health nurses" OR "community health nurse" OR "community health officers" OR "community health volunteer" OR "community health volunteers" OR "community health worker" OR "community health workers" OR "community IMCI" OR "community-oriented primary care" OR "community oriented primary care" OR "community volunteer" OR "community volunteers" OR "health extension workers" OR "integrated child development services" OR "Paramedical worker" OR "paramedical workers" OR "village development committee" OR "village development committees" OR "village health worker" OR "village health workers" OR "village health volunteer" OR "village health volunteers" OR "community-based nutrition programs" OR "community based nutrition programs" OR "community-based program" OR "community-based programs" OR "community-based programme" OR "community-based programmes" OR "community based programme" OR "community based programmes" OR "community involvement" OR "community participation" OR "community program" OR "community programs" OR "community programme" OR "community programmes")

Scopus

TITLE-ABS-KEY({maternal self-administration} OR {child welfare} OR {Community health workers} OR {Community Networks} OR {Community Health Aides} OR {Community Health Nursing} OR {Community Medicine} OR {community network} OR {community networks} OR {community health aide} OR {community health aides} OR {community health nursing} OR {community medicine} OR {community health nurses} OR {community health nurse} OR {community health officers} OR {community health volunteer} OR {community health volunteers} OR {community health worker} OR {community health workers} OR {community IMCI} OR {community-oriented primary care} OR {community oriented primary

care} OR {community volunteer} OR {community volunteers} OR {health extension workers} OR {integrated child development services} OR {Paramedical worker} OR {paramedical workers} OR {village development committee} OR {village development committees} OR {village health worker} OR {village health workers} OR {village health volunteer} OR {village health volunteers} OR {community-based nutrition programs} OR {community based nutrition programs} OR {community-based program} OR {community-based programs} OR {community-based programme} OR {community-based programmes} OR {community based programme} OR {community based programmes} OR {community involvement} OR {community participation} OR {community program} OR {community programs} OR {community programme} OR {community programmes})

AND

TITLE-ABS-KEY({HIV} OR {AIDS} OR {Human immunodeficiency viruses} OR {HTLV-III} OR {Human Immunodeficiency Virus} OR {Acquired Immune Deficiency} OR {Acquired Immunodeficiency} OR {T Lymphotropic Virus Type III Infections} OR {T-Lymphotropic Virus Type III Infection} OR {acquired immunologic deficiency})

TITLE({developing country} OR {developing countries} OR {developing nation} OR {developing nations} OR {developing population} OR {developing populations} OR {developing world} OR {less developed country} OR {less developed countries} OR {less developed nation} OR {less developed nations} OR {less developed population} OR {less developed populations} OR {less developed world} OR {lesser developed country} OR {lesser developed countries} OR {lesser developed nation} OR {lesser developed nations} OR {lesser developed population} OR {lesser developed populations} OR {lesser developed world} OR {under developed country} OR {under developed countries} OR {under developed nation} OR {under developed nations} OR {under developed population} OR {under developed populations} OR {under developed world} OR {underdeveloped country} OR {underdeveloped countries} OR {underdeveloped nation} OR {underdeveloped nations} OR {underdeveloped population} OR {underdeveloped populations} OR {underdeveloped world} OR {middle income country} OR {middle income countries} OR {middle income nation} OR {middle income nations} OR {middle income population} OR {middle income populations} OR {low income country} OR {low income countries} OR {low income nation} OR {low income nations} OR {low income population} OR {low income populations} OR {lower income country} OR {lower income countries} OR {lower income nation} OR {lower income nations} OR {lower income population} OR {lower income populations} OR {underserved country} OR {underserved countries} OR {underserved nation} OR {underserved nations} OR {underserved population} OR {underserved populations} OR {underserved world} OR {under served country} OR {under served countries} OR {under served nation} OR {under served nations} OR {under served population} OR {under served populations} OR {under served world} OR {deprived country} OR {deprived countries} OR {deprived nation} OR {deprived nations} OR {deprived population} OR {deprived populations} OR {deprived world} OR {poor country} OR {poor countries} OR {poor nation} OR {poor nations} OR {poor population} OR {poor populations} OR {poor world} OR {poorer country} OR {poorer countries} OR {poorer nation} OR {poorer nations} OR {poorer population} OR {poorer populations} OR {poorer world} OR {developing economy} OR {developing economies} OR {less developed economy} OR {less developed economies} OR {lesser developed

economy} OR {lesser developed economies} OR {under developed economy} OR {under developed economies} OR {underdeveloped economy} OR {underdeveloped economies} OR {middle income economy} OR {middle income economies} OR {low income economy} OR {low income economies} OR {lower income economy} OR {lower income economies} OR {low gdp} OR {low gnp} OR {low gross domestic} OR {low gross national} OR {lower gdp} OR {lower gnp} OR {lower gross domestic} OR {lower gross national} OR {lmic} OR {lmics} OR {third world} OR {lami country} OR {lami countries} OR {transitional country} OR {transitional countries} OR {Africa} OR {Asia} OR {Caribbean} OR {West Indies} OR {South America} OR {Latin America} OR {Central America} OR {Atlantic Islands} OR {Commonwealth of Independent States} OR {Pacific Islands} OR {Indian Ocean Islands} OR {Eastern Europe} OR {Afghanistan} OR {Albania} OR {Algeria} OR {Angola} OR {Antigua} OR {Barbuda} OR {Argentina} OR {Armenia} OR {Armenian} OR {Aruba} OR {Azerbaijan} OR {Bahrain} OR {Bangladesh} OR {Barbados} OR {Benin} OR {Byelarus} OR {Byelorussian} OR {Belarus} OR {Belorussian} OR {Belorussia} OR {Belize} OR {Bhutan} OR {Bolivia} OR {Bosnia} OR {Herzegovina} OR {Hercegovina} OR {Botswana} OR {Brasil} OR {Brazil} OR {Bulgaria} OR {Burkina Faso} OR {Burkina Fasso} OR {Upper Volta} OR {Burundi} OR {Urundi} OR {Cambodia} OR {Khmer Republic} OR {Kampuchea} OR {Cameroon} OR {Cameroons} OR {Cameron} OR {Camerons} OR {Cape Verde} OR {Central African Republic} OR {Chad} OR {Chile} OR {China} OR {Colombia} OR {Comoros} OR {Comoro Islands} OR {Comores} OR {Mayotte} OR {Congo} OR {Zaire} OR {Costa Rica} OR {Cote d'Ivoire} OR {Ivory Coast} OR {Croatia} OR {Cuba} OR {Cyprus} OR {Czechoslovakia} OR {Czech Republic} OR {Slovakia} OR {Slovak Republic} OR {Djibouti} OR {French Somaliland} OR {Dominica} OR {Dominican Republic} OR {East Timor} OR {East Timur} OR {Timor Leste} OR {Ecuador} OR {Egypt} OR {United Arab Republic} OR {El Salvador} OR {Eritrea} OR {Estonia} OR {Ethiopia} OR {Fiji} OR {Gabon} OR {Gabonese Republic} OR {Gambia} OR {Gaza} OR {Georgia Republic} OR {Georgian Republic} OR {Ghana} OR {Gold Coast} OR {Greece} OR {Grenada} OR {Guatemala} OR {Guinea} OR {Guam} OR {Guiana} OR {Guyana} OR {Haiti} OR {Honduras} OR {Hungary} OR {India} OR {Maldives} OR {Indonesia} OR {Iran} OR {Iraq} OR {Isle of Man} OR {Jamaica} OR {Jordan} OR {Kazakhstan} OR {Kazakh} OR {Kenya} OR {Kiribati} OR {Korea} OR {Kosovo} OR {Kyrgyzstan} OR {Kirghizia} OR {Kyrgyz Republic} OR {Kirghiz} OR {Kirgizstan} OR {Lao PDR} OR {Laos} OR {Latvia} OR {Lebanon} OR {Lesotho} OR {Basutoland} OR {Liberia} OR {Libya} OR {Lithuania} OR {Macedonia} OR {Madagascar} OR {Malagasy Republic} OR {Malaysia} OR {Malaya} OR {Malay} OR {Sabah} OR {Sarawak} OR {Malawi} OR {Nyasaland} OR {Mali} OR {Malta} OR {Marshall Islands} OR {Mauritania} OR {Mauritius} OR {Agalega Islands} OR {Melanesia} OR {Mexico} OR {Micronesia} OR {Middle East} OR {Moldova} OR {Moldovia} OR {Moldovian} OR {Mongolia} OR {Montenegro} OR {Morocco} OR {Ifni} OR {Mozambique} OR {Myanmar} OR {Myanma} OR {Burma} OR {Namibia} OR {Nepal} OR {Netherlands Antilles} OR {New Caledonia} OR {Nicaragua} OR {Niger} OR {Nigeria} OR {Northern Mariana Islands} OR {Oman} OR {Muscat} OR {Pakistan} OR {Palau} OR {Palestine} OR {Panama} OR {Paraguay} OR {Peru} OR {Philippines} OR {Philipines} OR {Phillipines} OR {Phillippines} OR {Poland} OR {Portugal} OR {Puerto Rico} OR {Romania} OR {Rumania} OR {Roumania} OR {Russia} OR {Russian} OR {Rwanda} OR {Ruanda} OR {Saint Kitts} OR {St Kitts} OR {Nevis} OR {Saint Lucia} OR {St Lucia} OR {Saint Vincent} OR {St Vincent} OR {Grenadines} OR {Samoa} OR {Samoan Islands} OR {Navigator Island} OR {Navigator Islands} OR {Sao Tome} OR

{Saudi Arabia} OR {Senegal} OR {Serbia} OR {Montenegro} OR {Seychelles} OR {Sierra Leone} OR {Slovenia} OR {Sri Lanka} OR {Ceylon} OR {Solomon Islands} OR {Somalia} OR {Sudan} OR {Suriname} OR {Surinam} OR {Swaziland} OR {Syria} OR {Syrian} OR {Tajikistan} OR {Tadzhikistan} OR {Tadjikistan} OR {Tadzhik} OR {Tanzania} OR {Thailand} OR {Togo} OR {Togolese Republic} OR {Tonga} OR {Trinidad} OR {Tobago} OR {Tunisia} OR {Turkey} OR {Turkmenistan} OR {Turkmen} OR {Tuvalu} OR {Uganda} OR {Ukraine} OR {Uruguay} OR {USSR} OR {Soviet Union} OR {Union of Soviet Socialist Republics} OR {Uzbekistan} OR {Uzbek} OR {Vanuatu} OR {New Hebrides} OR {Venezuela} OR {Vietnam} OR {Viet Nam} OR {West Bank} OR {Yemen} OR {Yugoslavia} OR {Zambia} OR {Zimbabwe} OR {Rhodesia} OR {Caribbean Region} OR {Central America} OR {Baltic States} OR {Republic of Belarus} OR {French Guiana} OR {Independent State of Samoa})

CINAHL

MH:"Human immunodeficiency virus" OR MH:"acquired immune deficiency syndrome" OR MH:"hiv infections"
